# Distinguishing Competing Mechanistic Manifolds for C(acyl)–N Functionalization by a Ni/*N*-Heterocyclic Carbene Catalyst System

**DOI:** 10.1021/jacsau.3c00283

**Published:** 2023-08-21

**Authors:** Kaycie R. Malyk, Vivek G. Pillai, William W. Brennessel, Roberto Leon Baxin, Elliot S. Silk, Daniel T. Nakamura, C. Rose Kennedy

**Affiliations:** University of Rochester, Department of Chemistry, Rochester, New York 14627, United States

**Keywords:** twisted amides, nickel, N-heterocyclic carbene, SIPr, mechanism

## Abstract

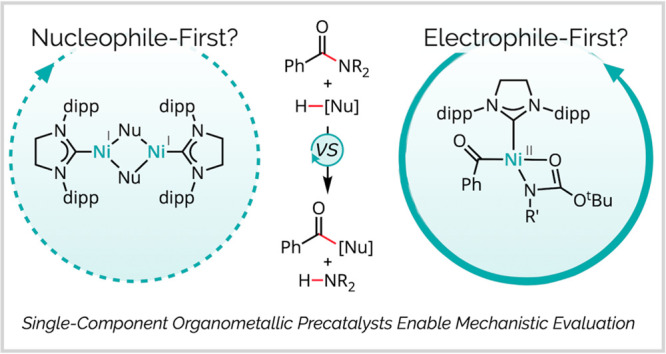

Carboxylic acid derivatives are appealing alternatives to organohalides as cross-coupling electrophiles for fine chemical synthesis due to their prevalence in biomass and bioactive small molecules as well as their ease of preparation and handling. Within this family, carboxamides comprise a versatile electrophile class for nickel-catalyzed coupling with carbon and heteroatom nucleophiles. However, even state-of-the-art C(acyl)–N functionalization and cross-coupling reactions typically require high catalyst loadings and specific substitution patterns. These challenges have proven difficult to overcome, in large part due to limited experimental mechanistic insight. In this work, we describe a detailed mechanistic case study of acylative coupling reactions catalyzed by the commonly employed Ni/SIPr catalyst system (SIPr = 1,3-bis(2,6-di-isopropylphenyl)-4,5-dihydroimidazol-2-ylidine). Stoichiometric organometallic studies, in situ spectroscopic measurements, and crossover experiments demonstrate the accessibility of Ni(0), Ni(I), and Ni(II) resting states. Although in situ precatalyst activation limits reaction efficiency, the low concentrations of active, SIPr-supported Ni(0) select for electrophile-first (closed-shell) over competing nucleophile-first (open-shell) mechanistic manifolds. We anticipate that the experimental insights into the nature and controlling features of these distinct pathways will accelerate rational improvements to cross-coupling methodologies involving pervasive carboxamide substrate motifs.

Over the past decade, tremendous progress has been reported in the use of carboxylic acid derivatives, such as carboxamides, as substrates for catalytic cross-coupling.^1−3^ Carboxamides are attractive alternatives to traditional organohalide electrophiles due to their relative abundance in biomass and drug-like molecules, ease of synthesis, and avoidance of halogenated waste streams.^4,5,8^ Typically, carboxamide C(acyl)–N functionalization methods rely on Ni catalysis due to the enhanced electropositivity of Ni (which facilitates activation of more polar C–O and C–N bonds) compared to conventional Pd cross-coupling catalysts (which favor reactions with more covalent C–halogen bonds).^9,10^ However, Ni readily undergoes both 1e^–^ and 2e^–^ processes, complicating method development and study.^9−11^

In contrast to conventional Pd-catalyzed cross-coupling reactions,^12−16^ the sequence of steps, identities of catalytically active species, and selectivity-determining factors remain ambiguous for Ni-catalyzed coupling reactions with carboxamide electrophiles. Studies of Ni/bisphosphine catalyst systems have begun to shed light on related coupling reactions with carboxylate ester and acyl fluoride electrophiles.^17−19^ However, complementary Ni/ *N*-heterocyclic carbene (NHC) catalyst systems remain comparatively understudied.^20,21^ This deficiency is noteworthy given the broad variety of acylative coupling reactions that rely on Ni/NHC precatalysts.^22,29−42^ Experimental characterization of catalytically relevant species and steps is thus essential to enable future rational improvements.

Herein, we report the systematic examination of C(acyl)–N functionalization reactions of carboxamide electrophiles catalyzed by the commonly employed combination of Ni and SIPr (SIPr = 1,3-bis(2,6-diisopropylphenyl)-4,5-dihydroimidazol-2-ylidine). We provide evidence for the accessibility and chemical competence of Ni(0), Ni(I), and Ni(II) resting states in such reactions, where the nucleophile identity and concentrations of C–N activated complexes play key roles in gating access to distinct off-cycle Ni(I) species. These findings shed light on the basis for catalyst inefficiencies and limited substrate compatibility, thereby providing the insights needed for rational development of next-generation methodologies with carboxamides and other carboxylic acid-derived electrophiles.

To probe the pathways involved in Ni-catalyzed C(acyl)–N functionalization, we elected to examine the landmark catalytic esterification and transamidation methodologies reported by Garg and co-workers in 2015 and 2016 (Scheme 1A).^22,29^ These catalytic methods achieve carbonyl-retentive C–heteroatom bond formation between alcohol or amine nucleophiles and *N*-functionalized benzamide electrophiles, which are commonly described as twisted amides.^43−45^ Although these methods are formally the equivalent of traditional acyl substitution chemistry, they proceed under comparatively mild conditions (room temperature to 80 °C) in the absence of strong Lewis or Brønsted acids. Although many cross-coupling reactions with C(acyl)–N electrophiles require highly twisted amide substrates, which exhibit enhanced electrophilicity due to disrupted n(N) → π*(CO) conjugation,^43,46−49^ the heteroatom coupling methods under study notably require only moderately activated substrates.

**Scheme 1:**
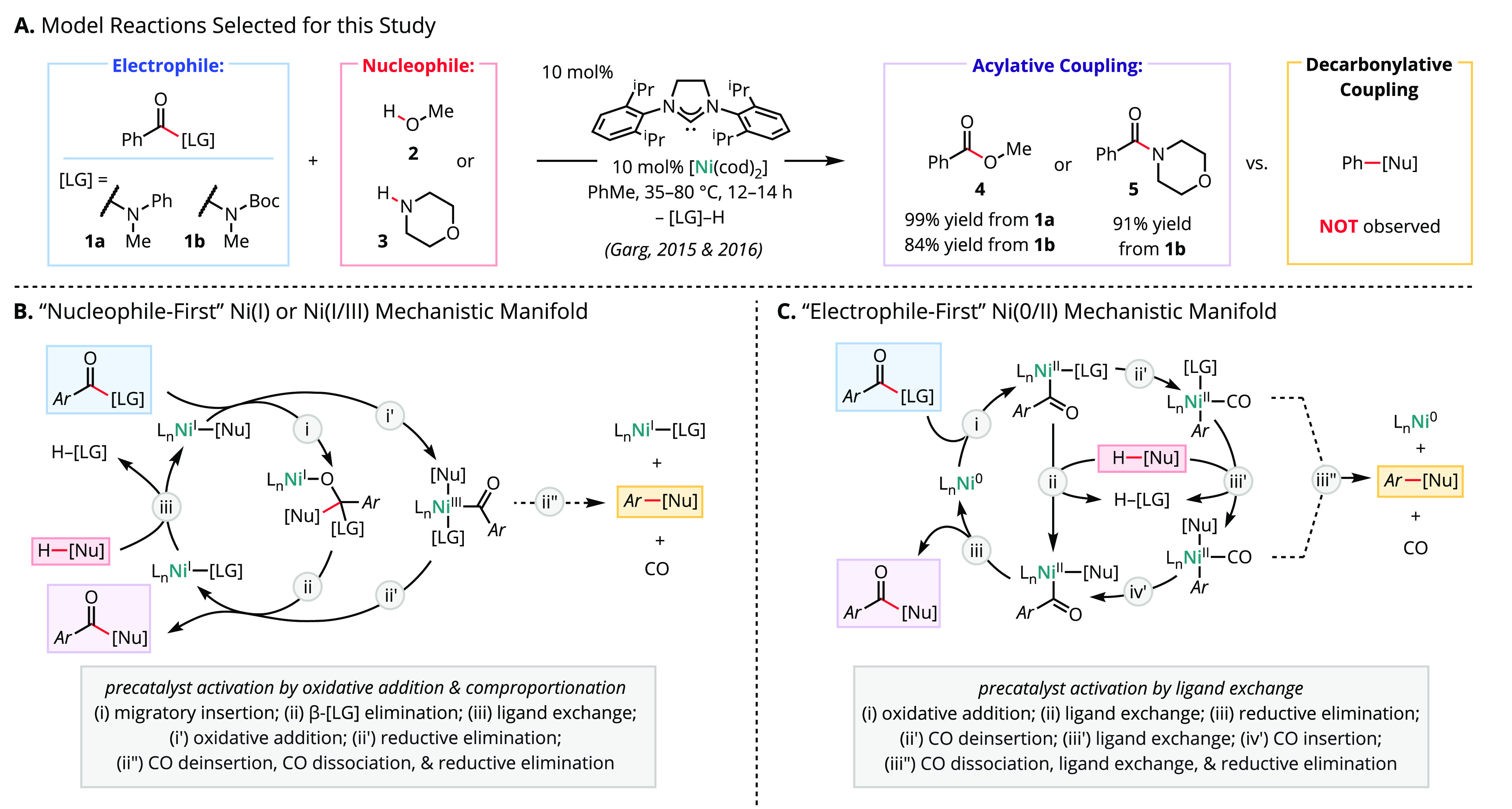
Model Reactions Selected for Evaluation of Competing Mechanistic Hypotheses Abbreviations: Ar = aryl; cod = 1,5-cyclooctadiene; [LG] = leaving group; [Nu] = nucleophile.

At the outset of our investigation, we identified two general mechanistic manifolds that could account for the observed carbonyl-retentive coupling. In a “nucleophile-first” manifold (Scheme 1B), initial formation of a Ni(I) nucleophile adduct would be followed by (i) migratory insertion with the twisted amide electrophile, (ii) β-elimination, and (iii) exchange of the leaving group for nucleophile to turn over the catalytic cycle. A variation of the “nucleophile-first” manifold could alternatively involve (i′) oxidative addition and (ii′) reductive elimination through a Ni (I/III) cycle, intercepting many of the same intermediates as the redox-neutral case above.

In an alternative “electrophile-first” manifold (Scheme 1 C) (i) initial C–N oxidative addition by Ni(0) would be followed by (ii) ligand exchange and (iii) carbon–nucleophile bond-forming reductive elimination. Despite the surprising preference for carbonyl-retentive reactivity (in contrast to the decarbonylative reactivity often noted with Ni(II) acyl complexes),^17−19^ the electrophile-first manifold is generally invoked, with computational studies supporting its energetic feasibility.^21,22^ However, no conclusive experimental validation has been disclosed, and to the best of our knowledge, no head-to-head comparison with alternative mechanistic hypotheses (such as the nucleophile-first case) has been conducted. Notably, kinetics alone cannot distinguish between the two manifolds, which may feature rate laws with analogous forms depending on the rate-determining step.^27^ Differentiating these pathways instead requires the direct identification of catalytically active intermediates.

In light of these mechanistic ambiguities, we first set out to identify the composition/speciation and resting state(s) of nickel under catalytically relevant conditions. In analogy to in situ activation protocols, equimolar [Ni(cod)_2_] and SIPr were mixed in benzene-*d*_6_ (0.1 M) at room temperature (approximately 22 °C) and monitored by ^1^H NMR spectroscopy (Scheme 2A).^50^ Generation of [(SIPr)Ni(C_6_D_6_)] (**6**) was noted, but even after mixing for several hours, substantial amounts of [Ni(cod)_2_] and SIPr remained in solution ([SIPr]:**6** = [Ni(cod)_2_]:[cod]/2 = 2:1). Although [Ni(SIPr)_2_] has previously been suggested as the primary catalyst resting state,^22^ it was not detected when employing a 1:1 ratio of [Ni] and ligand. Under catalytic conditions (10 mol % [Ni(cod)_2_]; 10 mol % SIPr) in the presence of amide **1a** and methanol (**2**), the low concentrations of **6** generated in situ were depleted but substantial [Ni(cod)_2_] and SIPr remained even after several hours, comprising an off-cycle catalyst resting state (Scheme 2B).^51^ In control experiments, neither [Ni(cod)]_2_ nor SIPr alone catalyzed the acylative couplings under investigation. As such, these findings are consistent with inefficient formation of **6** or another active catalyst, necessitating the relatively high loadings (≥10 mol %) typically required for these transformations.

**Scheme 2:**
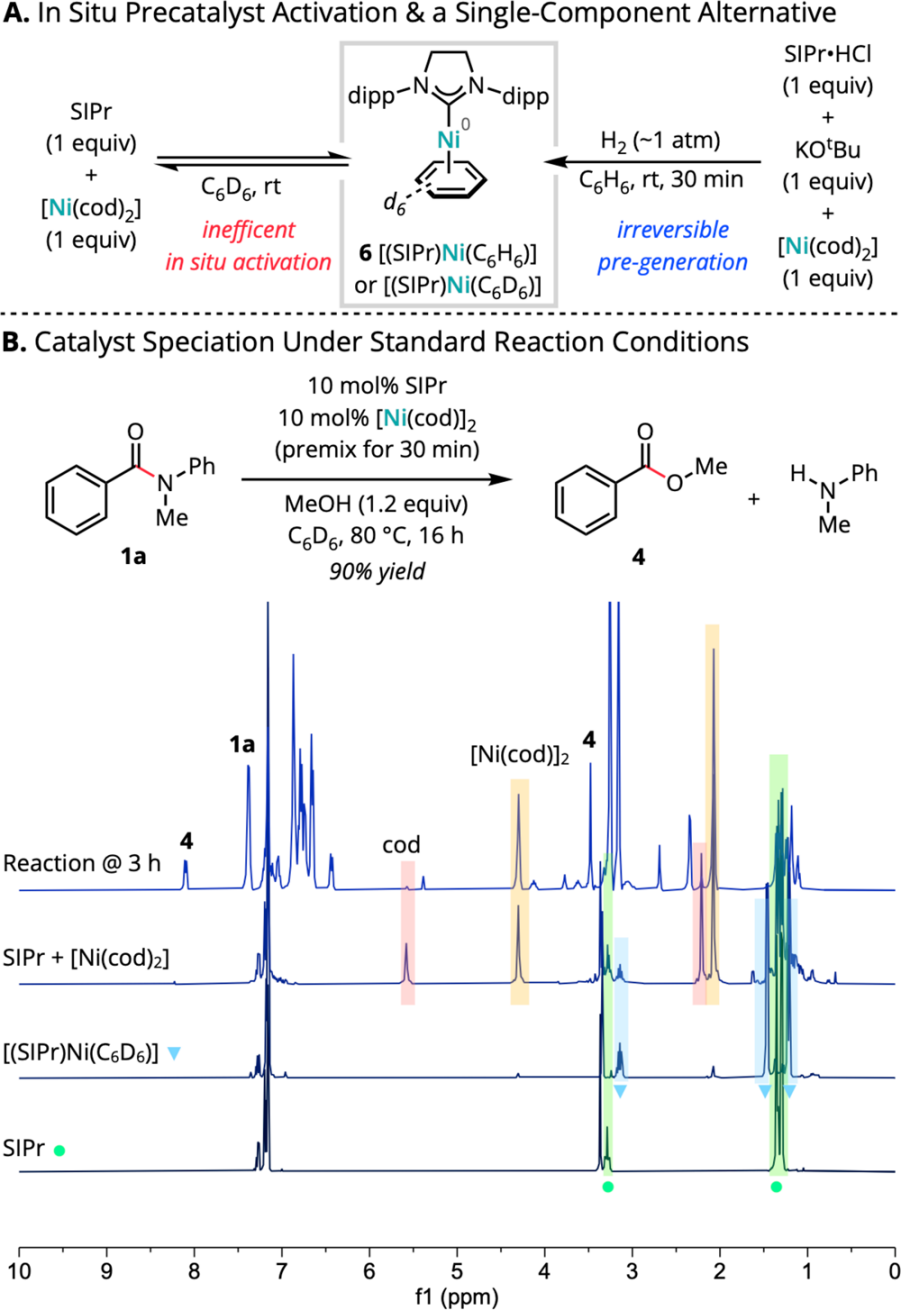
In Situ Precatalyst Activation Is Inefficient Abbreviations: dipp = 2,6-diisopropylphenyl; cod = 1,5-cyclooctadiene

To better assess the identities and catalytic competencies of the nickel-containing species generated in situ, we deemed it essential to work directly with single-component precatalysts. Following modification of conditions reported previously, the combination of [Ni(cod)_2_], SIPr·HCl, and KO^t^Bu in benzene under H_2_ pressure (∼1 atm, to effect the hydrogenation of 1,5-cyclooctadiene) afforded direct access to **6** (Scheme 2A).^52^ Reaction time-course experiments support the high activity of single-component **6** compared to the in situ precatalyst generation protocol (Supporting Information).

Hartwig and co-workers demonstrated previously that treating **6** with phenol resulted in the formation of nickel(I) phenoxide dimer [(SIPr)Ni(OPh)]_2_ (**7a**) via sequential oxidative addition and comproportionation (Scheme 3 A).^53−55^ Although phenols are poor substrates for the nickel-catalyzed esterification, presumably due to the low thermodynamic driving force for formation of the corresponding phenyl esters, we postulated that **7a** could act as a shunt into the nucleophile-first mechanistic manifold through ligand exchange to access on-cycle Ni(I) nucleophile adducts. Consistent with this hypothesis, using **7a** (5 mol % dimer, 10 mol % [Ni]) in place of SIPr/[Ni(cod)_2_] under otherwise standard conditions resulted in formation of ester **4** (22% yield), albeit with reduced conversion relative to standard conditions (Scheme 3B).

**Scheme 3:**
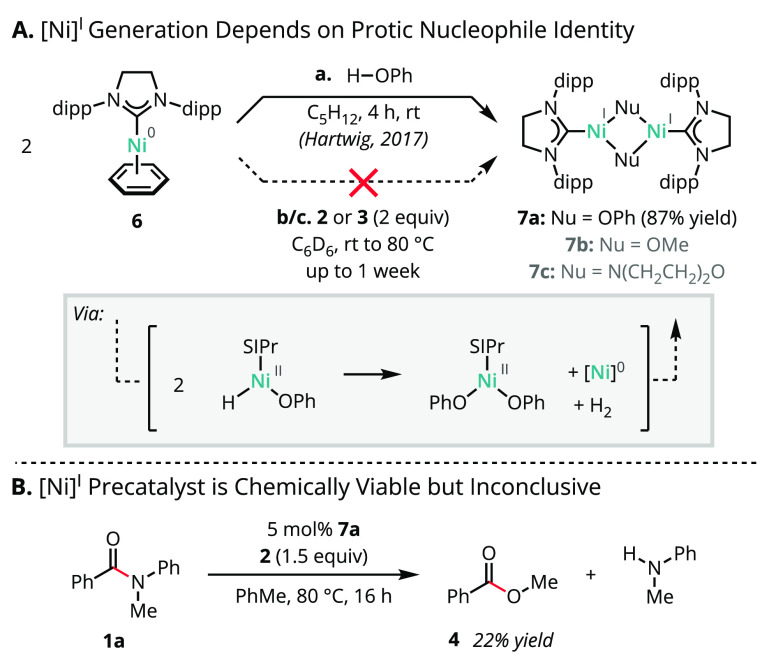
Access to and Viability of Catalytically Relevant Nickel(I) Complexes

We thus sought to determine whether the alcohol and amine nucleophiles applied for the model reactions could, in analogy to phenol, promote access to nickel(I).^56,57^ Mixing **6** with **2** or **3** (2.0 equiv) in benzene-*d*_6_ at room temperature up to 80 °C afforded negligible reactivity with no new species detected by ^1^H NMR until eventual decomposition of [(SIPr)Ni(C_6_D_6_)] (Scheme 3A).^58,59^ Taken together, these results suggest that oxidative addition into the stronger, less acidic O–H/N–H bonds of unactivated alcohols or amines is negligible in catalytically relevant arene solvents. However, these findings did not rule out the possibility of accessing Ni(I) species from alternative Ni(II) sources generated in situ (see below).

We next assessed the reactivity of single-component Ni(0) precatalyst **6** toward representative twisted amide substrates across a range of amidicities (Scheme 4 A).^44,48,60^ Amides **1b**–**d** underwent clean conversion to the corresponding SIPr-supported Ni(II) acyl products, which were isolated in 43–81% yield and characterized by SC-XRD (Figure 1), confirming their composition and connectivity. Although no oxidative addition products were detected with substrates lacking a carbamate directing group (e.g., **1a**), a crossover experiment between **1a** and fluorine-tagged substrate **1e** provided support for their kinetic accessibility (Scheme 4B).^61−63^

**Figure 1:**
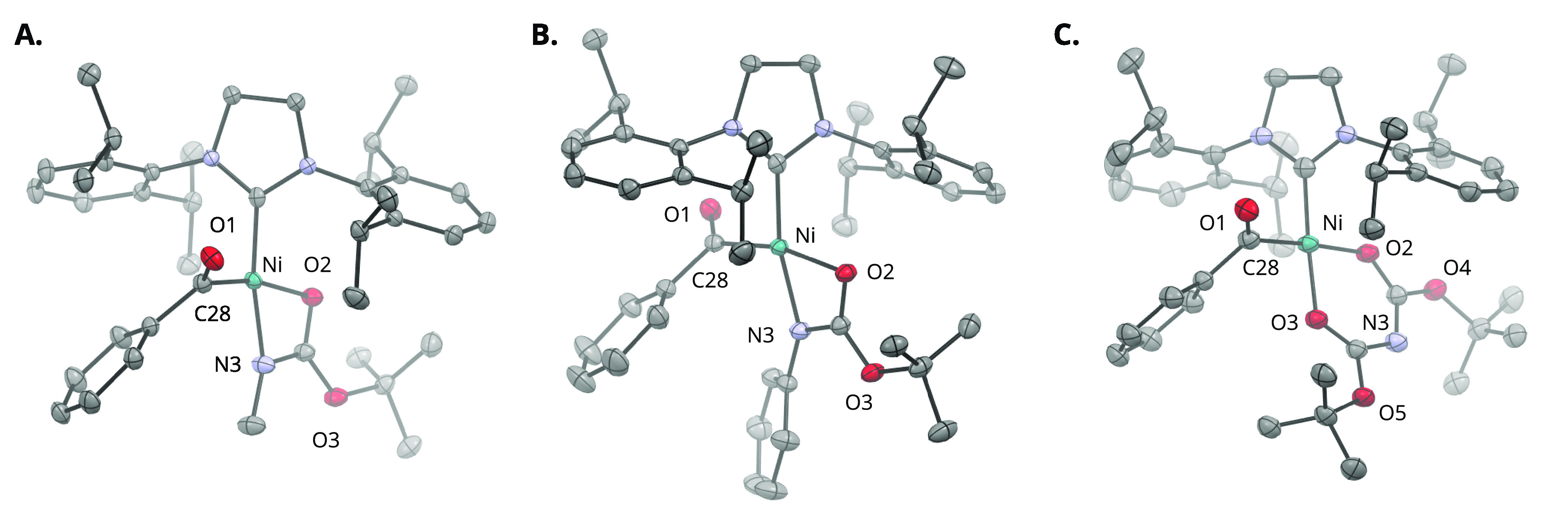
Solid-state structures of (A) **8b**, (B) **8c**, and (C) **8d** determined by SC-XRD. Thermal ellipsoids are depicted at 50% probability. H atoms and cocrystallized solvent molecules omitted for clarity. Color code: C, charcoal; N, blue; O, red; Ni, teal.

**Scheme 4:**
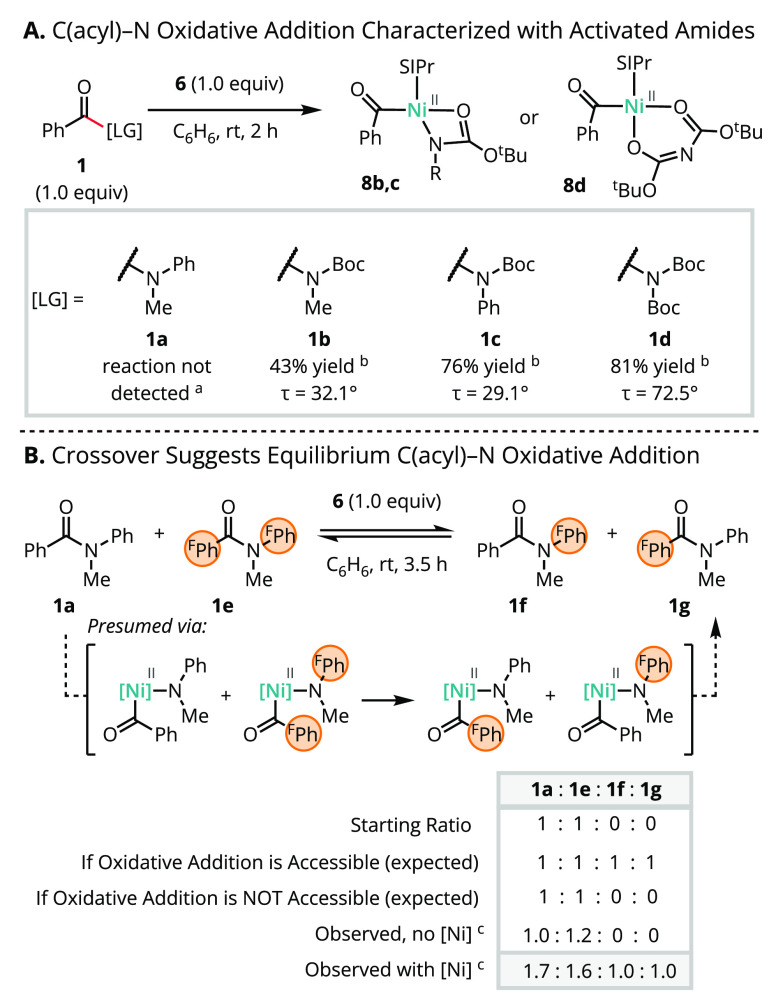
Kinetic Accessibility and Catalytic Relevance of C(acyl)–N Oxidative Addition Up to 80 °C. Isolated yields validated by integration of diagnostic ^1^H NMR resonances relative to 1,3,5-trimethoxybenzene as an internal standard. Winkler–Dunitz Distortion twist angles (τ) were taken from refs (44−46). Relative integration of diagnostic signals detected by analytical gas chromatography.

The solid-state structures for **8b**–**d** determined by SC-XRD (Figure 1) exhibited chelation by the carbamate directing group in either a κ^2^-N,O or κ^2^-O,O arrangement, resulting in distorted-square-planar coordination geometries (**8b**, τ_4_ = 0.17; **8c**, τ_4_ = 0.19; **8d**, τ_4_ = 0.10).^64^ In complexes **8b**,**c**, the NHC was found to be mutually *cis* to the benzoyl group, *cis* to the carbamate O, and *trans* to N in an arrangement minimizing steric interference with the bulky ligand wingtip substituents. While the geometric parameters for **8b**,**c** were near-identical, complex **8d** demonstrated a six-membered chelate with the two carbamate directing groups. Although such chelates have been described for phosphine-based catalyst systems,^65^ computational studies of NHC-based catalyst systems have instead invoked three-centered oxidative addition with disagreement over the role of directing-group assistance or chelation post-oxidative addition.^21,28^ Other chelating groups are expected to perform similarly; see the Supporting Information for the oxidative addition product derived from a 2-pyridyl substituted benzamide.

We next evaluated the chemical and catalytic competencies of these well-defined Ni(II) acyl complexes for acylative coupling model reactions. Even at room temperature, complexes **8b**,**c** react with excess nucleophile in benzene-*d*_6_ to yield acyl coupling products **4** (37% yield from **8b**) and **5** (30% yield from **8b**) within 24 h. Regeneration of **6** was observed, but no other organometallic intermediates were detected under these conditions. Using **8c** (10 mol %) in place of [Ni(cod)_2_]/SIPr under otherwise standard conditions similarly resulted in clean formation of product **4** (90% yield).

The chemical and catalytic competence of the Ni(II) acyl species supports the viability of the electrophile-first mechanistic manifold. However, an alternative providing access to Ni(I) and the nucleophile-first mechanistic manifold could not be excluded. In this case, reversible reductive elimination from the Ni(II) acyl species would enable Ni(0)–Ni(II) comproportionation. To evaluate the feasibility of this latter possibility, we examined the reactivity between **6** and the Ni(II) acyl complex **8c** in C_6_H_6_ at 80 °C (Scheme 5). The ^1^H NMR spectrum obtained after 5 h revealed complete consumption of both Ni complexes and the formation of at least two new species, including major components with paramagnetically shifted resonances. Further characterization of the resulting species by X-band electron paramagnetic resonance (EPR) spectroscopy in THF glass at 10 K afforded two sets of rhombic signals (simulated as *g*_A_ = [2.92, 2.38, 2.01], *g*_B_ = [2.54, 2.37, 2.01]). However, deploying this mixture of species as the precatalyst under otherwise standard conditions (i) did not promote any productive formation of product **4** using substrate **1a** and (ii) resulted in decreased yield and selectivity for **4** using substrate **1c**. In the latter case, products **9** and **10** resulting from the attack of the carbamoyl activating group were formed. In light of these findings, we hypothesize that high concentrations of Ni(II) derived from oxidative addition with carbamate-activated amides can lead to competitive rates of Ni(I) generation under catalytic conditions. This observation thus accounts for the high variability of the “optimal” leaving group substitution pattern determined empirically for the broad range of C(acyl)–N functionalization methodologies.^22,29−42^

**Scheme 5:**
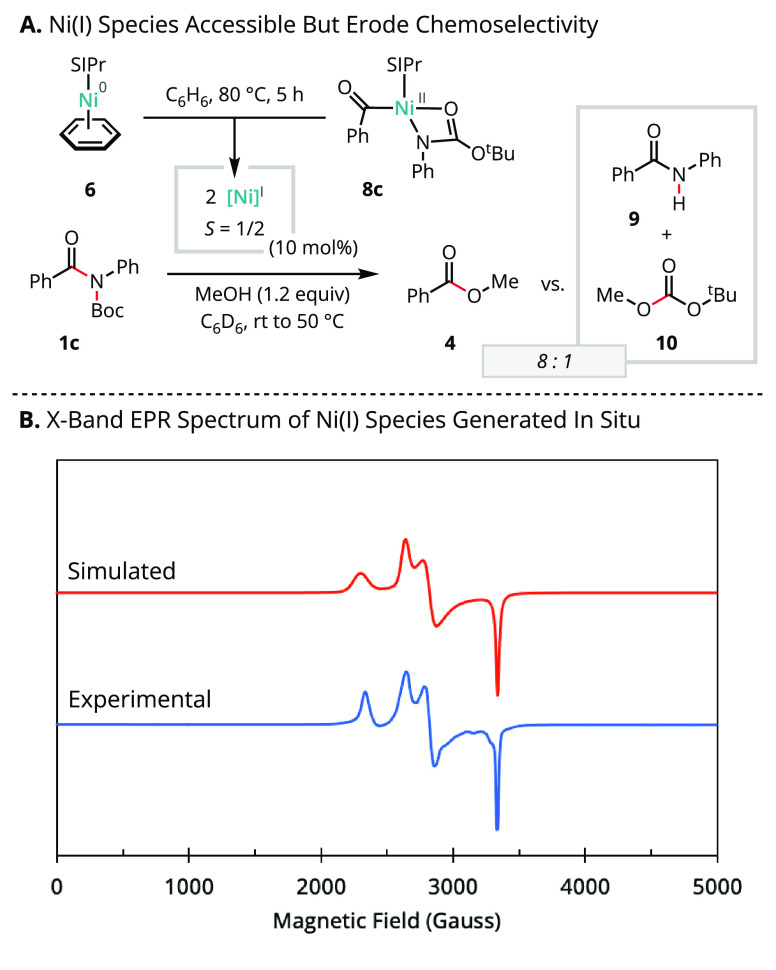
Evidence for Comproportionation of Amide Oxidative Addition Products

Taken together, these stoichiometric organometallic studies, in situ spectroscopic measurements, and crossover experiments provide evidence for the accessibility of Ni(0), Ni(II), *and* Ni(I) complexes under catalytically relevant conditions. Our work not only provides the first unambiguous experimental validation of the presumptive electrophile-first mechanistic manifold but also supports the viability of a competing nucleophile-first mechanistic manifold that induces counterproductive chemoselectivity.^66^ Notably, these distinctions could not be discerned from computational studies examining only closed-shell Ni(0/II) processes.^21−28^ Given the increasing prevalence of 1e^–^ processes in a broad variety of Ni-catalyzed reactions,^10^ we anticipate that these findings will provide a nuanced understanding to enable new and more efficient coupling reactions involving abundant acyl electrophiles.

## Supporting Information Available

Experimental details, compound characterization data, and results (PDF)
Crystallographic data for **8b** (CCDC 2264311), **8c** (CCDC 2264313), **8d** (CCDC 2264312), and **8i** (CCDC 2285470) (CIF)

## Supplementary Material

au3c00283_si_001.pdf

au3c00283_si_002.cif

## References

[ref1] Dander J. E.; Garg N. K. Breaking Amides using Nickel Catalysis. ACS Catal. 2017, 7, 1413–1423. 10.1021/acscatal.6b03277. 28626599 PMC5473294

[ref2] Takise R.; Muto K.; Yamaguchi J. Cross-coupling of aromatic esters and amides. Chem. Soc. Rev. 2017, 46, 5864–5888. 10.1039/C7CS00182G. 28685781

[ref3] Boit T. B.; Bulger A. S.; Dander J. E.; Garg N. K. Activation of C-O and C-N Bonds Using Non-Precious-Metal Catalysis. ACS Catal. 2020, 10, 12109–12126. 10.1021/acscatal.0c03334. 33868770 PMC8049354

[ref4] For example, carboxamides are found in over 50% of small-molecule pharmaceuticals, and amidation is the most common reaction class employed in medicinal chemistry settings. See refs (5−8).

[ref5] Walters W. P.; Green J.; Weiss J. R.; Murcko M. A. What Do Medicinal Chemists Actually Make? A 50-Year Retrospective. J. Med. Chem. 2011, 54, 6405–6416. 10.1021/jm200504p. 21755928

[ref6] Roughley S. D.; Jordan A. M. The Medicinal Chemist’s Toolbox: An Analysis of Reactions Used in the Pursuit of Drug Candidates. J. Med. Chem. 2011, 54, 3451–3479. 10.1021/jm200187y. 21504168

[ref7] Ertl P.; Altmann E.; McKenna J. M. The Most Common Functional Groups in Bioactive Molecules and How Their Popularity Has Evolved over Time. J. Med. Chem. 2020, 63, 8408–8418. 10.1021/acs.jmedchem.0c00754. 32663408

[ref8] Bhutani P.; Joshi G.; Raja N.; Bachhav N.; Rajanna P. K.; Bhutani H.; Paul A. T.; Kumar R. U.S. FDA Approved Drugs from 2015-June 2020: A Perspective. J. Med. Chem. 2021, 64, 2339–2381. 10.1021/acs.jmedchem.0c01786. 33617716

[ref9] Tasker S. Z.; Standley E. A.; Jamison T. F. Recent advances in homogeneous nickel catalysis. Nature 2014, 509, 299–309. 10.1038/nature13274. 24828188 PMC4344729

[ref10] Diccianni J. B.; Diao T. N. Mechanisms of Nickel-Catalyzed Cross-Coupling Reactions. Trends Chem. 2019, 1, 830–844. 10.1016/j.trechm.2019.08.004.

[ref11] The redox versatility also introduces opportunities for innovation, and a parallel area of vibrant research takes advantage of the unique 1-electron chemistry at nickel.

[ref12] Thomas A. A.; Denmark S. E. Pre-transmetalation intermediates in the Suzuki-Miyaura reaction revealed: The missing link. Science 2016, 352, 329–332. 10.1126/science.aad6981. 27081068

[ref13] Thomas A. A.; Wang H.; Zahrt A. F.; Denmark S. E. Structural, Kinetic, and Computational Characterization of the Elusive Arylpalladium(II)boronate Complexes in the Suzuki-Miyaura Reaction. J. Am. Chem. Soc. 2017, 139, 3805–3821. 10.1021/jacs.6b13384. 28266847 PMC7784246

[ref14] Hartwig J. F. Electronic Effects on Reductive Elimination To Form Carbon-Carbon and Carbon-Heteroatom Bonds from Palladium(II) Complexes. Inorg. Chem. 2007, 46, 1936–1947. 10.1021/ic061926w. 17348724

[ref15] Hartwig J. F.; Collman J. P. Organotransition metal chemistry: from bonding to catalysis; University Science Books: 2010; Vol. 1, pp 890–902.

[ref16] D'Alterio M. C.; Casals-Cruanas E.; Tzouras N. V.; Talarico G.; Nolan S. P.; Poater A. Mechanistic Aspects of the Palladium-Catalyzed Suzuki-Miyaura Cross-Coupling Reaction. Chem. Eur. J. 2021, 27, 13481–13493. 10.1002/chem.202101880. 34269488 PMC8518397

[ref17] Lalloo N.; Brigham C. E.; Sanford M. S. Mechanism-Driven Development of Group 10 Metal-Catalyzed Decarbonylative Coupling Reactions. Acc. Chem. Res. 2022, 55, 3430–3444. 10.1021/acs.accounts.2c00496. 36382937 PMC9764028

[ref18] Guo L.; Rueping M. Decarbonylative Cross-Couplings: Nickel Catalyzed Functional Group Interconversion Strategies for the Construction of Complex Organic Molecules. Acc. Chem. Res. 2018, 51, 1185–1195. 10.1021/acs.accounts.8b00023. 29652129

[ref19] Guo L.; Rueping M. Transition-Metal-Catalyzed Decarbonylative Coupling Reactions: Concepts, Classifications, and Applications. Chem. Eur. J. 2018, 24, 7794–7809. 10.1002/chem.201704670. 29757465

[ref20] Although several computational studies have been reported,^21−25^ experimental validation has been much more limited.^26−28^

[ref21] Wang H.; Zhang S.-Q.; Hong X. Computational studies on Ni-catalyzed amide C-N bond activation. Chem. Commun. 2019, 55, 11330–11341. 10.1039/C9CC05763C. 31468046

[ref22] Hie L.; Nathel N. F. F.; Shah T. K.; Baker E. L.; Hong X.; Yang Y. F.; Liu P.; Houk K. N.; Garg N. K. Conversion of amides to esters by the nickel-catalysed activation of amide C-N bonds. Nature 2015, 524, 79–83. 10.1038/nature14615. 26200342 PMC4529356

[ref23] Liu L. L.; Chen P.; Sun Y.; Wu Y.; Chen S.; Zhu J.; Zhao Y. Mechanism of Nickel-Catalyzed Selective C-N Bond Activation in Suzuki-Miyaura Cross-Coupling of Amides: A Theoretical Investigation. J. Org. Chem. 2016, 81, 11686–11696. 10.1021/acs.joc.6b02093. 27809510

[ref24] Xu Z.-Y.; Yu H.-Z.; Fu Y. Mechanism of Nickel-Catalyzed Suzuki-Miyaura Coupling of Amides. Chem. Asian J. 2017, 12, 1765–1772. 10.1002/asia.201700313. 28481443

[ref25] Chu C.-q.; Dang L. Esterification of Aryl and Alkyl Amides Enabled by Tailor-Made and Proposed Nickel Catalyst: Insights from Theoretical Investigation. J. Org. Chem. 2018, 83, 5009–5018. 10.1021/acs.joc.8b00160. 29620893

[ref26] Hu J.; Zhao Y.; Liu J.; Zhang Y.; Shi Z. Nickel-Catalyzed Decarbonylative Borylation of Amides: Evidence for Acyl C-N Bond Activation. Angew. Chem., Int. Ed. 2016, 55, 8718–8722. 10.1002/anie.201603068. 27258597

[ref27] Weires N. A.; Caspi D. D.; Garg N. K. Kinetic Modeling of the Nickel-Catalyzed Esterification of Amides. ACS Catal. 2017, 7, 4381–4385. 10.1021/acscatal.7b01444. 28713644 PMC5504487

[ref28] Zhong H.; Egger D.; Gasser V.; Finkelstein P.; Keim L.; Seidel M.; Trapp N.; Morandi B. Skeletal Metalation of Lactams through Carbonyl-to-Nickel Exchange Logic. ChemRxiv, 2023. 10.1038/s41467-023-40979-3 PMC10465567 37644031

[ref29] Baker E. L.; Yamano M. M.; Zhou Y. J.; Anthony S. M.; Garg N. K. A two-step approach to achieve secondary amide transamidation enabled by nickel catalysis. Nat. Commun. 2016, 7, 11554 10.1038/ncomms11554. 27199089 PMC4876455

[ref30] Hie L.; Baker E. L.; Anthony S. M.; Desrosiers J. N.; Senanayake C.; Garg N. K. Nickel-Catalyzed Esterification of Aliphatic Amides. Angew. Chem., Int. Ed. 2016, 55, 15129–15132. 10.1002/anie.201607856. PMC5161497 27813308

[ref31] Simmons B. J.; Weires N. A.; Dander J. E.; Garg N. K. Nickel-Catalyzed Alkylation of Amide Derivatives. ACS Catal. 2016, 6, 3176–3179. 10.1021/acscatal.6b00793. 32257581 PMC7111456

[ref32] Weires N. A.; Baker E. L.; Garg N. K. Nickel-catalysed Suzuki-Miyaura coupling of amides. Nat. Chem. 2016, 8, 75–79. 10.1038/nchem.2388. 26673267

[ref33] Ito Y.; Nakatani S.; Shiraki R.; Kodama T.; Tobisu M. Nickel-Catalyzed Addition of C-C Bonds of Amides to Strained Alkenes: The 1,2-Carboaminocarbonylation Reaction. J. Am. Chem. Soc. 2022, 144, 662–666. 10.1021/jacs.1c09265. 35005886

[ref34] Zheng Y.-L.; Xie P.-P.; Daneshfar O.; Houk K. N.; Hong X.; Newman S. G. Direct Synthesis of Ketones from Methyl Esters by Nickel-Catalyzed Suzuki-Miyaura Coupling. Angew. Chem., Int. Ed. 2021, 60, 13476–13483. 10.1002/anie.202103327. 33792138

[ref35] Ben Halima T.; Masson-Makdissi J.; Newman S. G. Nickel-Catalyzed Amide Bond Formation from Methyl Esters. Angew. Chem., Int. Ed. 2018, 57, 12925–12929. 10.1002/anie.201808560. 30113123

[ref36] Zheng Y.-L.; Newman S. G. Methyl Esters as Cross-Coupling Electrophiles: Direct Synthesis of Amide Bonds. ACS Catal. 2019, 9, 4426–4433. 10.1021/acscatal.9b00884.

[ref37] Zheng Y.-L.; Newman S. G. Nickel-Catalyzed Domino Heck-Type Reactions Using Methyl Esters as Cross-Coupling Electrophiles. Angew. Chem., Int. Ed. 2019, 58, 18159–18164. 10.1002/anie.201911372. 31574201

[ref38] Walker J. A.; Vickerman K. L.; Humke J. N.; Stanley L. M. Ni-Catalyzed Alkene Carboacylation via Amide C-N Bond Activation. J. Am. Chem. Soc. 2017, 139, 10228–10231. 10.1021/jacs.7b06191. 28708388

[ref39] Li J.-F.; Wang Y.-F.; Wu Y.-Y.; Liu W.-J.; Wang J.-W. Nickel-Catalyzed Esterification of Amides Under Mild Conditions. Catal. Lett. 2020, 150, 874–880. 10.1007/s10562-019-02966-6.

[ref40] Buchspies J.; Rahman M. M.; Szostak M. Suzuki-Miyaura Cross-Coupling of Amides Using Well-Defined, Air- and Moisture-Stable Nickel/NHC (NHC = N-Heterocyclic Carbene) Complexes. Catalysts 2020, 10, 372 10.3390/catal10040372.

[ref41] Buchspies J.; Szostak M. Recent Advances in Acyl Suzuki Cross-Coupling. Catalysts 2019, 9, 53 10.3390/catal9010053.

[ref42] Li G.; Szostak M. Non-Classical Amide Bond Formation: Transamidation and Amidation of Activated Amides and Esters by Selective N-C/O-C Cleavage. Synthesis 2020, 52, 2579–2599. 10.1055/s-0040-1707101.

[ref43] Liu C. W.; Szostak M. Twisted Amides: From Obscurity to Broadly Useful Transition-Metal-Catalyzed Reactions by N-C Amide Bond Activation. Chem. Eur. J. 2017, 23, 7157–7173. 10.1002/chem.201605012. 27813178

[ref44] Meng G.; Zhang J.; Szostak M. Acyclic Twisted Amides. Chem. Rev. 2021, 121, 12746–12783. 10.1021/acs.chemrev.1c00225. 34406005 PMC9108997

[ref45] Gao P.; Rahman M. M.; Zamalloa A.; Feliciano J.; Szostak M. Classes of Amides that Undergo Selective N-C Amide Bond Activation: The Emergence of Ground-State Destabilization. J. Org. Chem. 2022, 10.1021/acs.joc.2c01094. 36054817

[ref46] Szostak R.; Shi S. C.; Meng G. R.; Lalancette R.; Szostak M. Ground-State Distortion in N-Acyl-tert-butyl-carbamates (Boc) and N-Acyl-tosylamides (Ts): Twisted Amides of Relevance to Amide N-C Cross-Coupling. J. Org. Chem. 2016, 81, 8091–8094. 10.1021/acs.joc.6b01560. 27480938

[ref47] Szostak R.; Meng G.; Szostak M. Resonance Destabilization in N-Acylanilines (Anilides): Electronically-Activated Planar Amides of Relevance in N-C(O) Cross-Coupling. J. Org. Chem. 2017, 82, 6373–6378. 10.1021/acs.joc.7b00971. 28590733

[ref48] Meng G. R.; Shi S. C.; Lalancette R.; Szostak R.; Szostak M. Reversible Twisting of Primary Amides via Ground State N-C(O) Destabilization: Highly Twisted Rotationally Inverted Acyclic Amides. J. Am. Chem. Soc. 2018, 140, 727–734. 10.1021/jacs.7b11309. 29240413

[ref49] The extent of activation or twisting is typically quantified using the Winkler–Dunitz distortion parameters including twist angle (τ) about the amide bond and nitrogen pyramidalization parameter (χ). See ref (43).

[ref50] Qualitatively similar results were obtained using toluene-*d*_8_ to mimic the preferred solvent in catalytic methodologies. As such, benzene-*d*_6_ was employed for further studies due to its lower cost and greater availability.

[ref51] For analogous experiments conducted with the combination of SIPr and [Ni(cod)_2_] in toluene-*d*_8_ the remaining [Ni(cod)_2_] is detected through the time required for full consumption of amide **1a** (see the Supporting Information).

[ref52] Hoshimoto Y.; Hayashi Y.; Suzuki H.; Ohashi M.; Ogoshi S. One-Pot, Single-Step, and Gram-Scale Synthesis of Mononuclear [(η^6^-arene)Ni(N-heterocyclic carbene)] Complexes: Useful Precursors of the Ni0-NHC Unit. Organometallics 2014, 33, 1276–1282. 10.1021/om500088p.

[ref53] Saper N. I.; Hartwig J. F. Mechanistic Investigations of the Hydrogenolysis of Diaryl Ethers Catalyzed by Nickel Complexes of N-Heterocyclic Carbene Ligands. J. Am. Chem. Soc. 2017, 139, 17667–17676. 10.1021/jacs.7b10537. 29116776 PMC11620758

[ref54] Bismuto A.; Müller P.; Finkelstein P.; Trapp N.; Jeschke G.; Morandi B. One to Find Them All: A General Route to Ni(I)-Phenolate Species. J. Am. Chem. Soc. 2021, 143, 10642–10648. 10.1021/jacs.1c03763. 34251813

[ref55] Lin C.-Y.; Power P. P. Complexes of Ni(i): a “rare” oxidation state of growing importance. Chem. Soc. Rev. 2017, 46, 5347–5399. 10.1039/C7CS00216E. 28675200

[ref56] Dodd N. A.; Bacsa J.; Sadighi J. P. Synthesis of a Nickel(I) alkoxide and related cation equivalents. Polyhedron 2021, 208, 115408 10.1016/j.poly.2021.115408.

[ref57] Lipschutz M. I.; Tilley T. D. Useful Method for the Preparation of Low-Coordinate Nickel(I) Complexes via Transformations of the Ni(I) Bis(amido) Complex K{Ni[N(SiMe_3_)(2,6-iPr_2_-C_6_H_3_)]_2_}. Organometallics 2014, 33, 5566–5570. 10.1021/om500849u. 25328273 PMC4195509

[ref58] Although new species were observed after heating (50–80 °C) in pentane, the catalytic relevance of the species resulting in noncoordinating solvent was unclear.

[ref59] Performing the analogous experiment with **2** in pentane at room temperature resulted in a color change (mahogany red to yellow-orange) and afforded a mixture of products detected by ^1^H NMR. Although recrystallization attempts were unsuccessful, the major components did not exhibit NMR features resembling nickel(I) alkoxides synthesized previously.^53,56^

[ref60] Treating the mixed precatalyst system generated in situ with BzN(Boc)_2_ (**1d**) resulted in burst-lag kinetics with fast consumption of [(SIPr)Ni(C_6_D_6_)] (within minutes) followed by slow consumption of [Ni(cod)_2_] and SIPr over the ensuing 24 h, coupled with line broadening and presumed decomposition of resulting products (see the Supporting Information).

[ref61] Haynes M. T. II; Liu P.; Baxter R. D.; Nett A. J.; Houk K. N.; Montgomery J. Dimer Involvement and Origin of Crossover in Nickel-Catalyzed Aldehyde-Alkyne Reductive Couplings. J. Am. Chem. Soc. 2014, 136, 17495–17504. 10.1021/ja508909u. 25401337 PMC4277774

[ref62] Zhang Y.; Wang Z.; Lamine W.; Xu S.; Li B.; Chrostowska A.; Miqueu K.; Liu S.-Y. Mechanism of Pd/Senphos-Catalyzed trans-Hydroboration of 1,3-Enynes: Experimental and Computational Evidence in Support of the Unusual Outer-Sphere Oxidative Addition Pathway. J. Org. Chem. 2023, 88, 2415–2424. 10.1021/acs.joc.2c02841. 36752741 PMC10162691

[ref63] The observation of crossover suggests that C–N oxidative addition is fast and reversible but thermodynamically uphill. As such, the equilibrium concentrations of the resulting nickel(II) acyl species are not detected by NMR or isolable under standard conditions.

[ref64] Yang L.; Powell D. R.; Houser R. P. Structural variation in copper(i) complexes with pyridylmethylamide ligands: structural analysis with a new four-coordinate geometry index, τ_4_. Dalton Trans. 2007, 955–964. 10.1039/B617136B. 17308676

[ref65] Xie P.-P.; Qin Z.-X.; Zhang S.-Q.; Hong X. Understanding the Structure-Activity Relationship of Ni-Catalyzed Amide C-N Bond Activation using Distortion/Interaction Analysis. ChemCatChem. 2021, 13, 3536–3542. 10.1002/cctc.202100672.

[ref66] A catalytic cycle is provided in the Supporting Information to synthesize these mechanistic findings and illustrate the competing reaction manifolds.

