# Supplementary material for: Distinguishing Competing Mechanistic Manifolds for C(acyl)–N Functionalization by a Ni/N-Heterocyclic Carbene Catalyst System
Source: JACS Au. 2023 Aug 21;3(9):2451–7. doi: 10.1021/jacsau.3c00283 (PMC10523494; doi:10.1021/jacsau.3c00283)
Supplement: Supplementary file 1 — au3c00283_si_001.pdf [file au3c00283_si_001.pdf]

Supporting Information

Distinguishing Competing Mechanistic Manifolds for C(acyl)–N Functionalization by a Ni/*N*-Heterocyclic Carbene Catalyst System

Kaycie R. Malyk[‡], Vivek G. Pillai[‡], William W. Brennessel, Roberto Leon Baxin, Elliot S. Silk, Daniel T. Nakamura, and C. Rose Kennedy*

Department of Chemistry, University of Rochester

Rochester, New York 14627, United States

* c.r.kennedy@rochester.edu

Table of Contents for the Supporting Information

1. Procedures, Materials, and Instrumentation S3

1.1 General Considerations S3

1.2 Materials S3

1.3 Instrumentation and Software S3

1.4 Abbreviations S4

2. Synthesis and Characterization of Amide Substrates S4

3. Synthesis and Characterization of Nickel Complexes S8

3.1 [(SIPr)Ni(η^6–C_6H_6)] (**6**) S8

3.2 General Procedure for Oxidative Addition of Twisted Amides S8

3.3 Attempted Oxidative Addition with Alternative Substrates S13

4. Catalytic Reactions S15

4.1 Catalytic Reactions with In Situ Precatalyst Generation S15

4.2 Catalytic Reactions with Single-Component (Pre-formed) Precatalysts S15

5. Crossover Experiments S17

6. Comproportionation Experiments S18

7. Reaction Time-course Experiments S21

7.1 General Procedures S21

7.2 Reaction Time-course at 80 °C S22

7.3 Reaction Time-course at 40 °C S25

8. SC-XRD Data S29

8.1 General Procedure S29

8.2 Solid-State Structure of **8b** S29

8.3 Solid-State Structure of **8c**•0.75 Et_2O S33

8.4 Solid-State Structure of **8d** S39

8.5 Solid-State Structure of **8i**•C_6H_{14} S43

9. Catalytic Cycle S48

10. References S49

1. Procedures, Materials, and Instrumentation

1.1 General Considerations

All air- and moisture-sensitive techniques were carried out using standard Schlenk technique on a Schlenk line or a high-vacuum line[1] or in an M. Braun glovebox containing an atmosphere of N_2. The glovebox was equipped with vacuum feed-throughs, a cold well, and a freezer for storing samples at –30 °C. Colors are described in comparison to the complete list of Prismacolor colored pencils.[2] Column chromatography was performed on SiliaFlash P60 (230–400 mesh) silica gel from SiliCycle using standard glass columns. Thin-layer chromatography (TLC) was performed using aluminum-backed plates pre-coated with silica gel and a fluorescent indicator for visualization upon UV irradiation.

1.2 Materials

Reagents were purchased in reagent grade from commercial suppliers and used without further purification unless described otherwise. Bis(cyclooctadiene) nickel [Ni(cod)$_2$] was purchased from Strem and stored at –30 °C in the glovebox. SIPr•HCl was prepared according to a reported literature procedure.[3] [(SIPr)Ni(η^6–C_6H_6)] **(6)** was synthesized according to a modified literature procedure.[4] [(SIPr)Ni(μ−OPh)]$_2$ dimer **7** was prepared as described previously.[5]

Solvents (acetonitrile, diethyl ether, *n*-pentane, tetrahydrofuran, and toluene) used for air- and moisture-sensitive manipulations were dried and deoxygenated by passage through an activated alumina column and stored over activated molecular sieves.[6, 7] Deuterated solvents used for NMR spectroscopy of air- and moisture-sensitive compounds were stirred over sodium (C_6D_6, THF-d_8) or calcium hydride (CD_3CN) and distilled prior to storage in the glovebox.

1.3 Instrumentation and Software

Nuclear Magnetic Resonance (NMR) and Electron Paramagnetic Resonance spectroscopies were performed at the University of Rochester, Department of Chemistry, Magnetic Resonance Facility. NMR spectra were recorded at 25 °C on a Bruker 400 or 500 Avance I spectrometer operating at: 400.13 or 500.20 MHz (^{1}H NMR), 100.25 or 125.78 MHz (^{13}C{^{1}H} NMR), or 376.43 MHz (^{19}F{^{1}H} NMR). Chemical shifts for ^{1}H and ^{13}C are reported in parts per million downfield from tetramethylsilane ($SiMe_4$) and are referenced in ppm relative to the NMR solvent according to literature values:[7] $\delta(^1H) = 7.16$, $\delta(^{13}C) = 128.1$ for C_6D_6.; $\delta(^1H) = 7.26$, $\delta(^{13}C) = 77.2$ for $CDCl_3$. Chemical shifts for ^{19}F are reported in parts per million downfield from neat $CFCl_3$ and are referenced in ppm relative to α,α,α-trifluorotoluene ($PhCF_3$) added as an internal standard. ^{1}H NMR data for diamagnetic substances are reported as follows: chemical shift, (multiplicity, coupling constant in Hz, integration) where s = singlet, d = doublet, t = triplet, q = quartet, m = multiplet, and br = broad. ^{13}C and ^{19}F NMR data for diamagnetic substances are reported as lists of chemical shifts. NMR spectra were processed using the MestReNova software suite. Electron paramagnetic resonance (EPR) spectra were recorded at 10 K, 9.38 GHz on a Bruker EMXplus spectrometer equipped with a 4119HS cavity and an Oxford ESR-900 helium flow cryostat. EPR spectra were fit using EasySpin in Matlab.[8]

Mass spectrometry was performed at the University of Rochester, Department of Chemistry, Instrumentation Facility. Liquid Chromatography Mass Spectrometry (LC-MS) data were collected

performed using Agilent Technology 1260 Infinity II LC system with an Advion Expression CMS detector using electrospray ionization (ESI). Gas chromatography Mass Spectrometry (GC-MS) data were collected on a Shimadzu GCMS-2010 using helium carrier gas and a ZB-XLB 0.25 mm x 30 m x 0.25 um (Phenomenex) column and EI (Electron Impact) ionization at 70 V and 60 uA emission current.

1.4 Abbreviations

Ar = aryl; Boc = tert-butyloxycarbonyl; Bz = benzoyl; cod = 1,5-cyclooctadiene; DMAP = 4-(dimethylamino)pyridine; EI = electron-impact ionization; EPR = electron paramagnetic resonance spectroscopy; ESI = electrospray ionization; HMDSO = hexamethyldisiloxane; IR = infrared spectroscopy; LRMS = low-resolution mass spectrometry; NHC = N-heterocyclic carbene; NMR = nuclear magnetic resonance spectroscopy; SC-XRD = single-crystal X-ray diffraction analysis; SIPr = 1,3-bis(2,6-diisopropylphenyl)-4,5-dihydro-imidazol-3-ium-2-ide; THF = tetrahydrofuran; UV/vis = ultraviolet-visible absorption spectroscopy.

2. Synthesis and Characterization of Amide Substrates

All twisted amide substrates were prepared with minor modifications to literature procedures. Amide **1a** was prepared as reported previously.[9]

General Procedure for Boc-Functionalization: An oven-dried round-bottom flask was charged with Boc_2O (1.5 – 2.5 equiv.), secondary or primary benzamide (16.5 mmol, 1.0 equiv.), and a magnetic stir bar. The flask was sealed, then evacuated and backfilled with N_2 for three cycles, after which MeCN (0.1 – 0.2 M) was added via cannula transfer. DMAP (0.244 g, 2.00 mmol, 0.12 equiv.) was added to the reaction mixture as a solid. The reaction was stirred at room temperature under N_2 flow and monitored by TLC. After 7–20 hours, the solvent was removed in vacuo and the desired product was isolated by flash silica gel column chromatography**.**

***Tert*-butyl benzoyl(methyl)carbamate (1b)** was synthesized following the representative procedure for Boc-functionalization of the corresponding secondary amide (0.991 g, 84%). Spectral data was in accordance with the literature.[10]

***Tert*-butyl benzoyl(phenyl)carbamate (1c)** was synthesized following the representative procedure for Boc-functionalization of the corresponding secondary amide (2.707 g, 91%). Spectral data was in accordance with the literature.[11]

***Tert*-butyl benzoyl(*tert*-butoxycarbonyl)carbamate (1d)** was synthesized following the representative procedure for Boc-functionalization of the corresponding primary amide (4.86 g, 92**%**). Spectral data was in accordance with the literature.[12]

4-Fluoro-*N*-(4-fluorophenyl)-*N*-methylbenzamide (1e) was synthesized using a procedure adapted from the synthesis of **1a**. An oven-dried round-bottom flask was charged with a magnetic stir bar, then evacuated and back-filled with N_2 for 3 cycles. Under N_2 flow, 4-fluoro-N-methylaniline (1.0 mL, 8.31 mmol, 1.0 equiv.), pyridine (25 mL, 0.33 M), and DCM (25 mL, 0.33 M) were added while stirring. The flask was cooled to 0 °C in an ice bath, and 4-fluorobenzoyl chloride (1.2 mL, 9.97 mmol, 1.2 equiv.) was added dropwise while stirring. The flask was warmed to room temperature and stirred under N_2 flow for 80 minutes to give a sepia/artichoke-colored mixture. The reaction mixture was diluted with EtOAc and washed with excess 1.0 M HCl (4x), then washed with brine (3x) and dried over Mg_2SO_4. The mixture was filtered and then concentrated in vacuo. Following purification by flash, silica-gel column chromatography (eluent: 30% EtOAc/70% hexanes) product **1e** was obtained as a sand-colored solid (0.842 g, 41%).

^{1}H NMR: (400 MHz, $CDCl_3$) δ 7.29 (t, J = 8.7, 6.0 Hz, 2H), 7.01 (dd, J = 8.5, 4.8 Hz, 2H), 6.94 (t, J = 8.5 Hz, 2H), 6.87 (t, J = 8.7 Hz, 2H), 3.46 (s, 3H)

^{13}C NMR: (101 MHz, $CDCl_3$) δ ^{13}C NMR (101 MHz, Chloroform-*d*) δ 169.7, 163.4 (d, J = 240.0 Hz), 160.9 (d, J = 236.7 Hz), 141.0 (d, J = 2.9 Hz), 131.8 (d, J = 3.4 Hz), 131.1 (d, J = 8.7 Hz), 128.6 (d, J = 8.5 Hz), 116.4 (d, J = 22.7 Hz), 115.1 (d, J = 21.8 Hz), 38.8

^{19}F{^{1}H} NMR: (376 MHz, $CDCl_3$) δ -109.7(m), -114.4.

LRMS (ESI): for $[M+H]^+$ = $C_{14}H_{12}F_2NO$, predicted *m/z* = 248.1, found *m/z* = 248.3

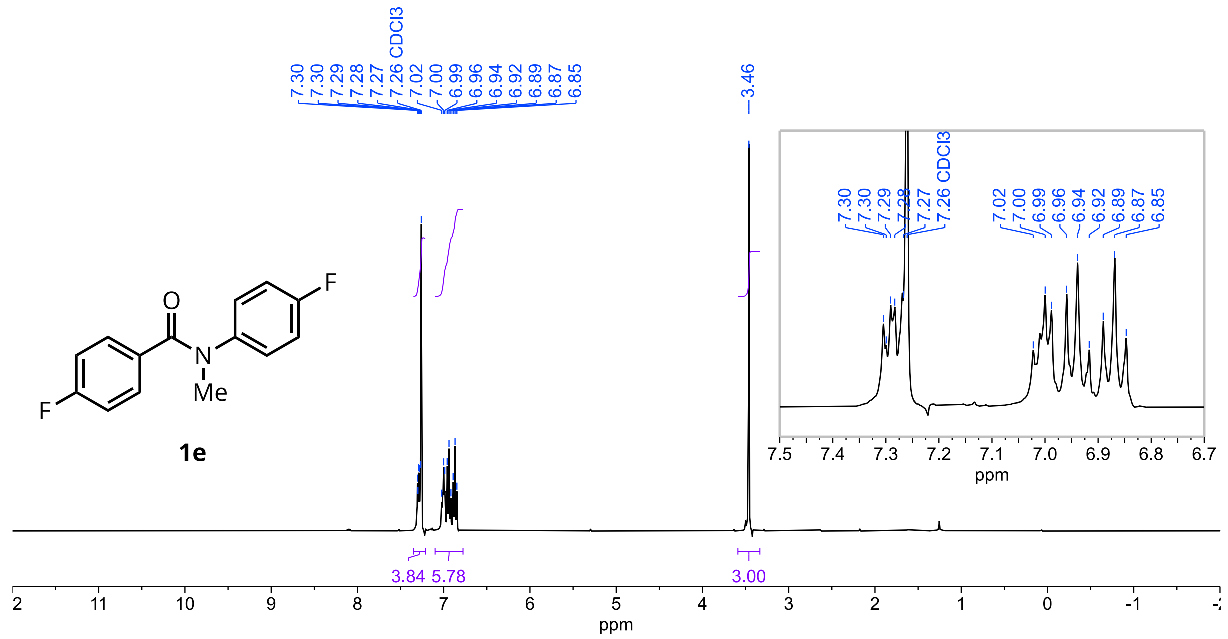

Figure S1. ^{1}H NMR (400 MHz, $CDCl_3$) spectrum of amide **1e.**

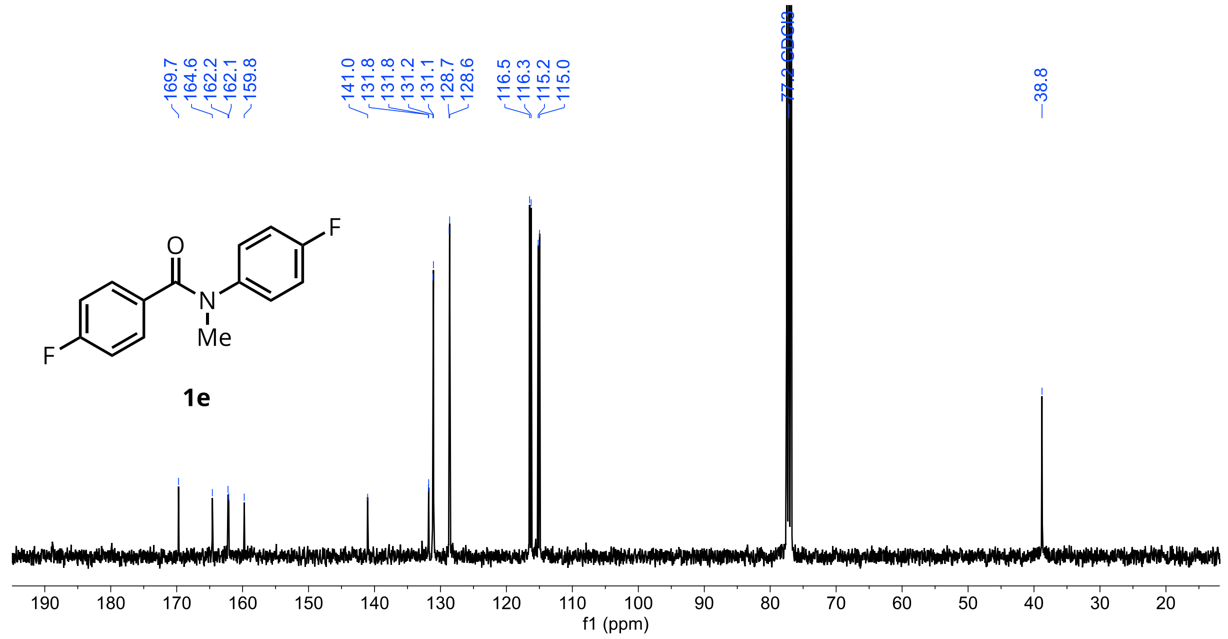

Figure S2. $^{13}C\{^{1}H\}$ NMR (101 MHz, $CDCl_3$) spectrum of amide **1e.**

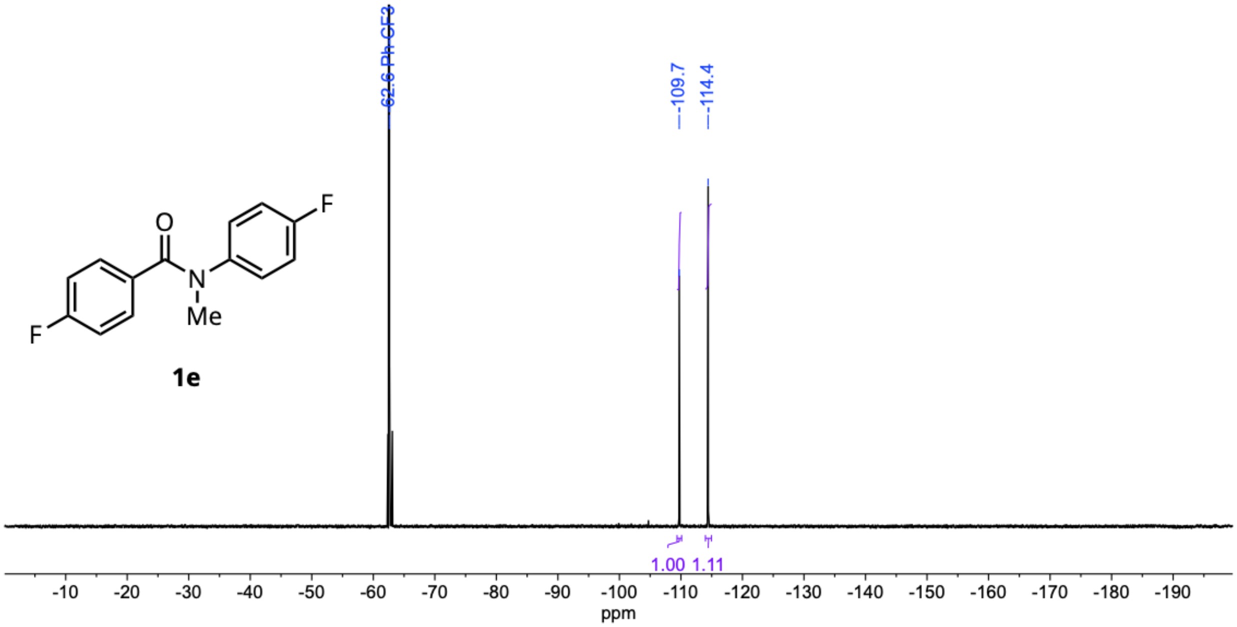

Figure S3. $^{19}F\{^{1}H\}$ NMR (376 MHz, $CDCl_3$) spectrum of amide **1e.**

3. Synthesis and Characterization of Nickel Complexes

3.1 [(SIPr)Ni(η^6–C_6H_6)] (6) was synthesized according to a modified literature procedure.[4] In a nitrogen-filled glovebox, three separate scintillation vials were prepared. Vial **A** was charged with Ni(cod)$_2$ (0.0185 g, 0.067 mmol, 1.0 equiv.). Vial **B** was charged with SIPr•HCl (0.0295 g, 0.07 mmol, 1.1 equiv.), and a magnetic stir bar. Vial **C** was charged with KO^{t}Bu (0.0078 g, 0.070 mmol, 1.0 equiv.) and 2 mL C_6H_6. Vial **C** was added to vial **B**, then rinsed with an additional 1 mL of C_6H_6. Vial **B** was stirred for ~ 5 min at room temperature, then vial **A** was added to vial **B**. The mixture was stirred, then transferred to a 100 mL solvent tube and sealed under N_2. The flask was brought outside of the glovebox and degassed through a freeze-pump-thaw sequence (3x). The flask was frozen in a liquid N_2 bath, then H_2 (2.4 atm) was added via vacuum gas transfer. The reaction mixture was thawed slowly, then returned to the glovebox and stirred for 30 minutes at room temperature under H_2 atmosphere. Upon completion, the mixture was filtered through celite with C_6H_6 (~ 12 mL) and concentrated in vacuo to a crimson lake-colored solid (0.0333 g, 94%). If necessary, the complex was purified by recrystallization from HMDSO. Spectral data were in accordance with the literature.[4,13]

3.2 General Procedure for Oxidative Addition of Twisted Amides: In a nitrogen-filled glovebox, a scintillation vial was charged with [(SIPr)Ni((η^6–C_6H_6)] (**6**) (0.040 g, 0.074 mmol, 1.0 equiv.), amide (**1**) (0.074 mmol, 1.0 equiv), and a magnetic stir bar. C_6H_6 (3 mL) was added, and the reaction was stirred at room temperature for 2 hours. After 2 hours, the mixture was concentrated in vacuo and washed 3 times with pentane, which was decanted to yield a yellow solid. The solid was dried in vacuo and characterized via NMR prior to recrystallization.

3.2.1 [Ni(SIPr)(benzoyl)((methyl)(*tert*-butoxycarbonyl)amide)] (8b) was synthesized according to the general procedure using amide **1b** (0.0106 g, 0.046 mmol). A yellow solid was obtained (0.046 g, 43%). Single crystals suitable for X-ray diffraction were obtained by recrystallization from heptane at –30 °C; see section 6 for structural data.

^{1}H NMR (400 MHz, C_6D_6) δ 8.00 (br. s, 2H), 7.22 – 7.03 (m, 6H), 6.96 (br. s, 3H), 3.72 (s, 4H), 3.45 (s, 4H), 1.79 (s, 3H), 1.73 (s, 6H), 1.71 (s, 6H), 1.25 (s, 6H), 1.23 (s, 6H), 1.08 (s, 9H)

^{13}C NMR (126 MHz, C_6D_6) δ 241.4, 212.0, 163.2, 147.7, 139.6, 137.0, 133.8, 124.7, 77.6, 54.1, 28.88, 28.85, 28.6, 26.6, 24.1

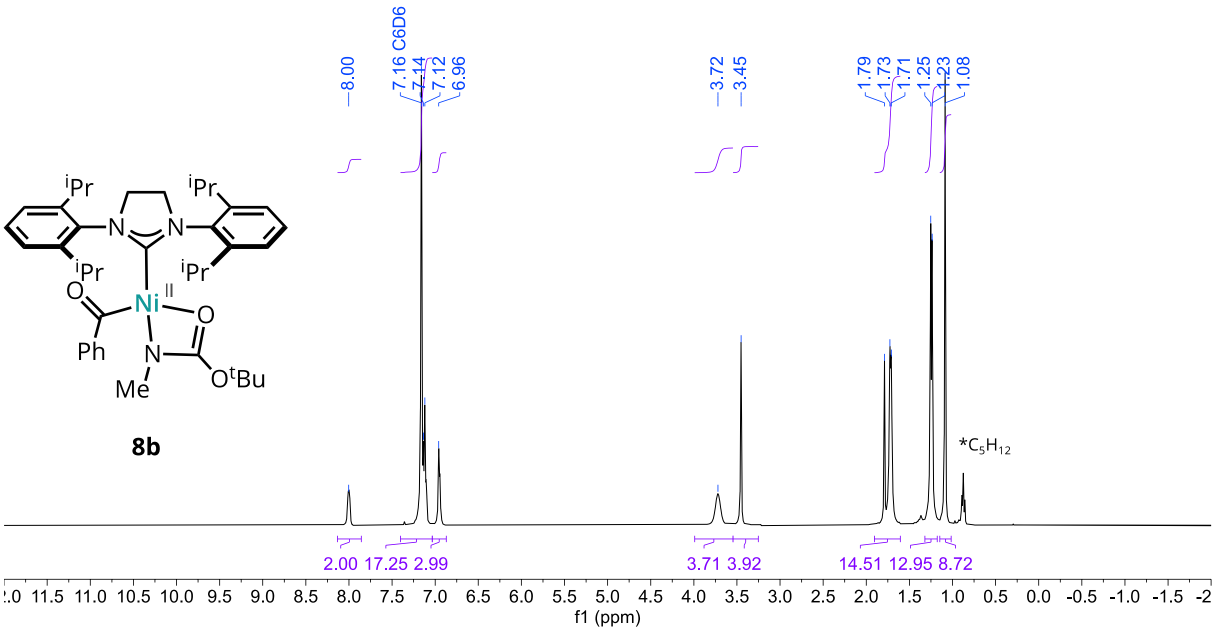

Figure S4. ^{1}H NMR (400 MHz, C_6D_6) spectrum of nickel complex **8b.**

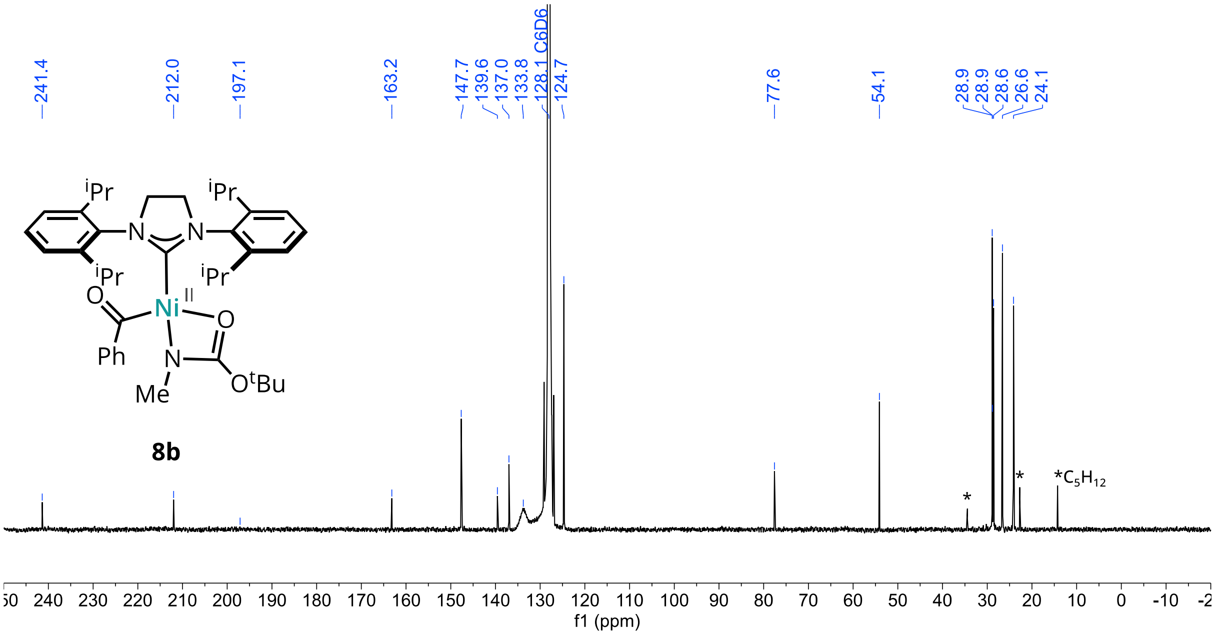

Figure S5. ^{13}C NMR (125 MHz, C_6D_6) spectrum of nickel complex **8b.**

3.2.2 [Ni(SIPr)(benzoyl)((phenyl)(*tert*-butoxycarbonyl)amide)] (8c) was synthesized according to the general procedure using amide **1c** (0.040 g, 0.076 mmol). A yellow solid was obtained (0.0424 g, 76%). Single crystals suitable for X-ray diffraction were obtained by recrystallization from Et_2O at –30 °C; see section 6 for structural data.

^{1}H NMR: (400 MHz, C_6D_6) δ 7.94 (d, J = 4.6 Hz, 2H), 7.09 (m, 5H), 6.78 (t, J = 7.0 Hz, 1H), 6.75 (t, J = 7.9, 7.0 Hz, 2H), 6.70 (d, J = 7.9 Hz, 2H), 6.54 (t, J = 7.0 Hz, 1H), 3.76 (br. s, 4H), 3.48 (s, 4H), 1.74 (br. s, 12H), 1.24 (s, 6H), 1.23 (s, 6H), 1.04 (s, 9H)

^{13}C NMR: (125 MHz, C_6D_6) δ 241.8, 211.1, 161.6, 147.6, 143.7, 138.3, 136.8, 129.2, 129.1, 127.5, 126.3, 124.9, 124.7, 121.4, 79.0, 54.2, 28.9, 28.4, 26.7, 24.1

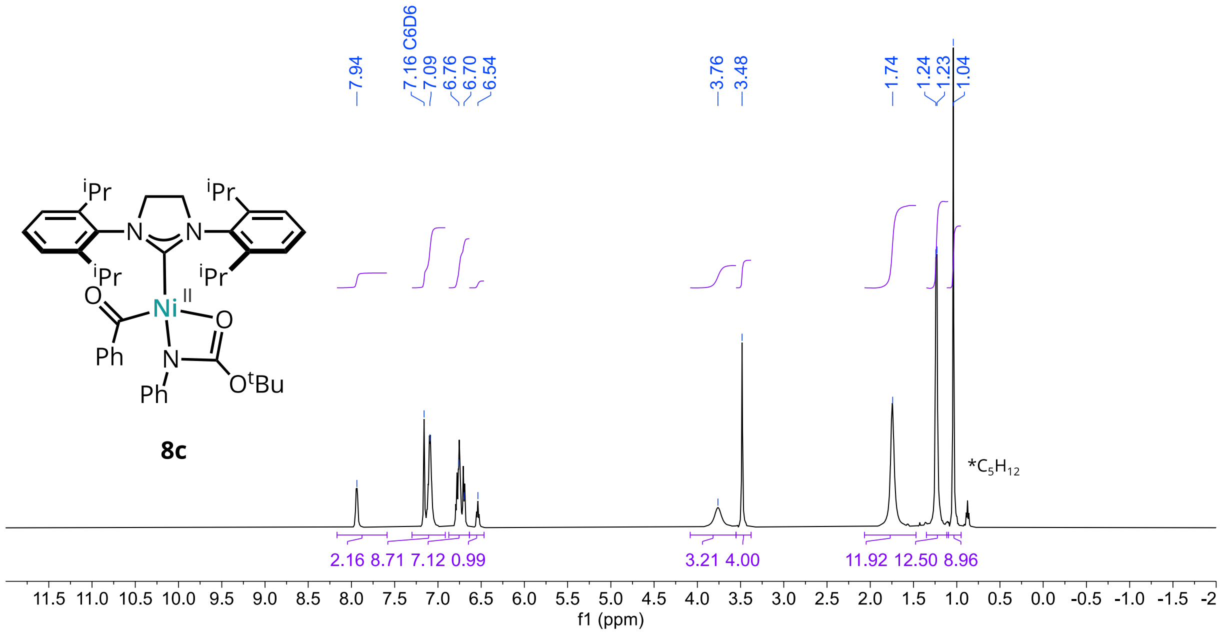

Figure S6. ^{1}H NMR (400 MHz, C_6D_6) spectrum of nickel complex **8c.**

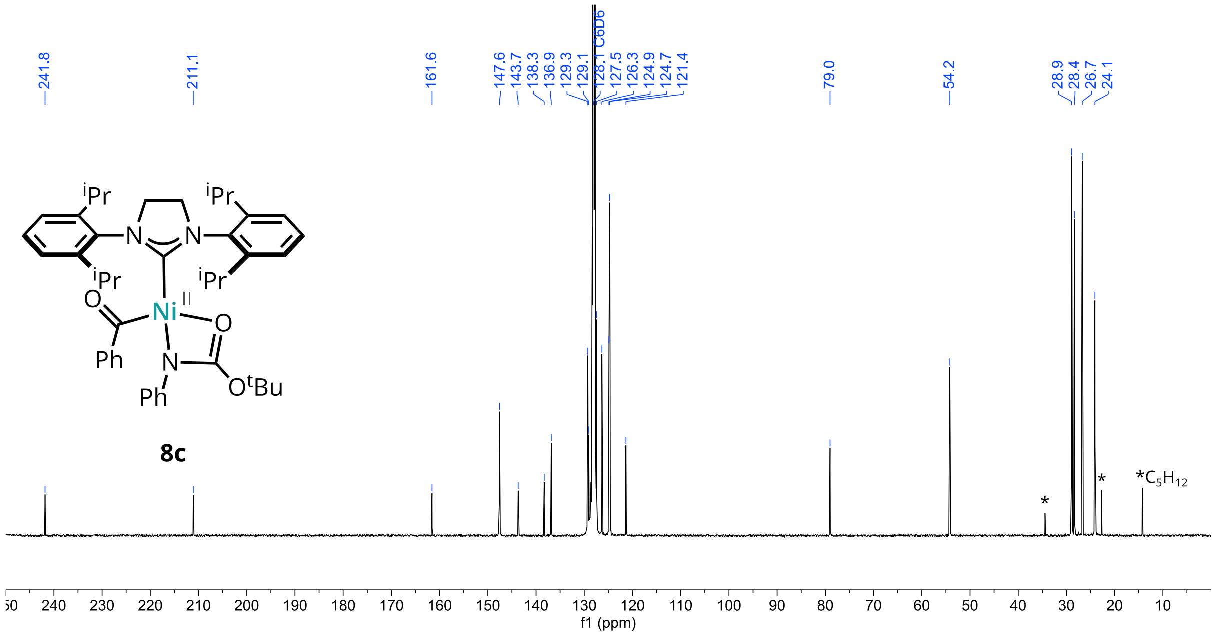

Figure S7. ^{13}C NMR (125 MHz, C_6D_6) spectrum of nickel complex **8c.**

3.2.3 [Ni(SIPr)(benzoyl)(bis(*tert*-butoxycarbonyl)amide)] (8d) was synthesized according to the general procedure using amide **1d** (0.0219 g, 0.074 mmol). A yellow solid was obtained (0.023 g, 81%). Single crystals suitable for X-ray diffraction were obtained by recrystallization from Et_2O at –30 °C; see section 6 for structural data.

^{1}H NMR: (400 MHz, C_6D_6) δ 8.33 (d, J = 6.4 Hz, 2H), 7.96 (br. s, 1H), 7.16 (m, 2H, overlaps with solvent), 7.02 (t, J = 7.4 Hz, 2H), 6.97 – 6.92 (m, 1H), 6.92 – 6.81 (m, 3H), 4.17 (br. s, 4H), 3.45 (s, 4H), 1.69 (br. s, 3H), 1.37 (s, 3H), 1.30 – 1.14 (m, 15H, overlapping signals), 1.07 (s, 18H), 0.75 (s, 3H)

^{13}C NMR (101 MHz, C_6D_6) δ 210.4, 208.4, 166.6, 165.7, 147.8, 140.2, 137.0, 136.9, 136.3, 129.3, 129.25, 129.21, 129.19, 127.1, 126.6, 124.7, 78.9, 78.5, 54.0, 53.9, 30.2, 28.9, 28.6, 27.93, 27.8, 27.5, 27.0, 24.0

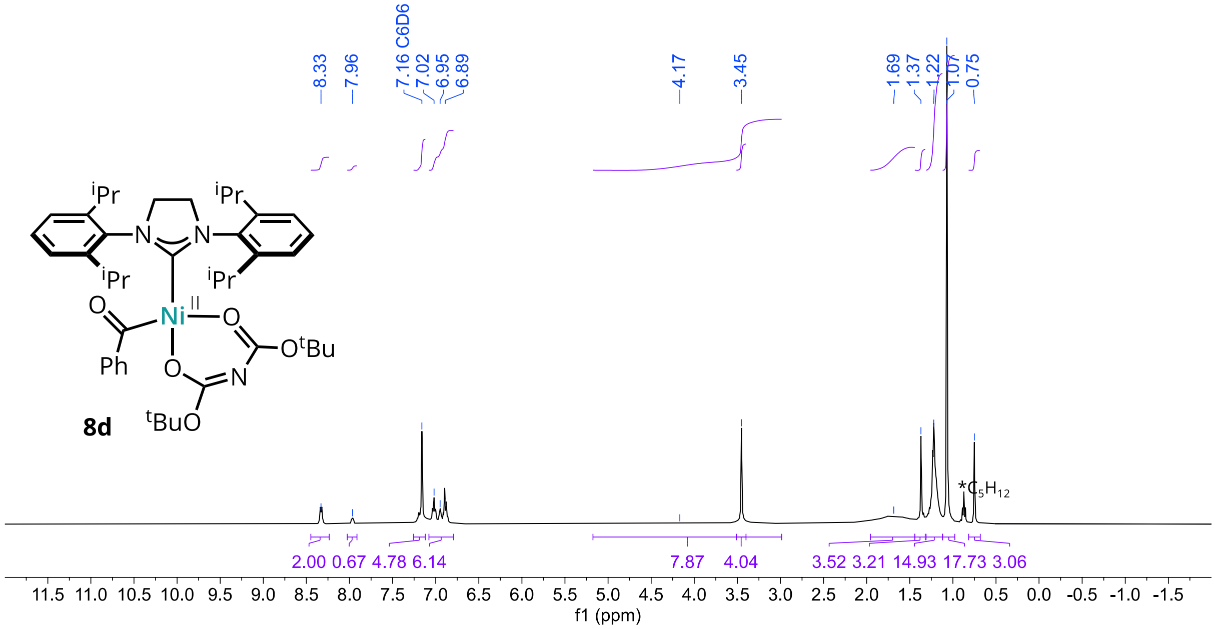

Figure S8. ^{1}H NMR (400 MHz, C_6D_6) spectrum of nickel complex **8d.**

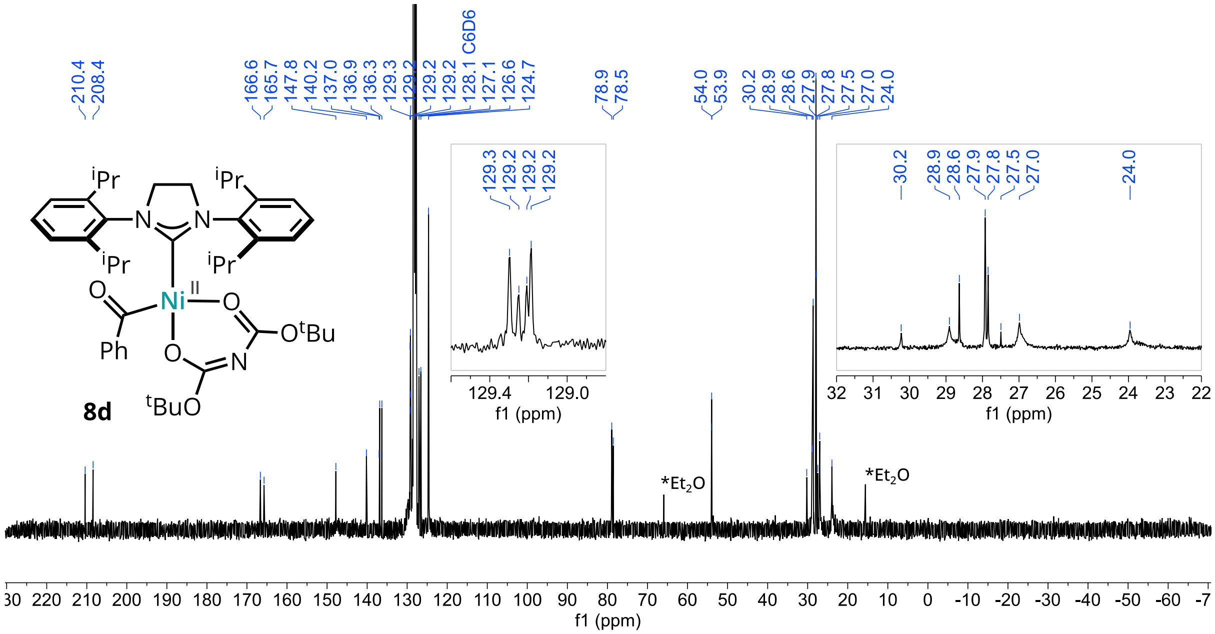

Figure S9. ^{1}H NMR (101 MHz, C_6D_6) spectrum of nickel complex **8d.**

3.3 Attempted Oxidative Addition with Alternative Substrates: In a nitrogen-filled glovebox, two separate 1-dram vials were charged with (A) [(SIPr)Ni((η^6–C_6H_6)] (**6**) (1.0 equiv, 0.09 mmol) and (B) amide (1.0 equiv., 0.09 mmol). The [Ni] complex from vial A was transferred to vial B as using small portions of C_6D_6 (0.7 mL total). All contents of vial B were transferred to a JYoung NMR tube using a glass pipet, which was then sealed and removed from the glovebox. The JYoung NMR tube was kept at room temperature (~22 °C), monitoring periodically by ^{1}H NMR spectroscopy. If little-to-no conversion was observed after 3 hours, the vial was transferred to a 50 °C in a bead bath, and ^{1}H NMR spectra was collected periodically at 25 °C. Conversion was monitored through the disappearance of diagnostic signals corresponding to **1** and **6** correlated with the appearance of new resonances. If new products were detected as a substantial portion of the crude material, the JYoung tube was returned to the glovebox and the contents transferred to a scintillation via. The mixture was concentrated in vacuo and washed 3 times with pentane, which was decanted to yield a yellow solid. The solid was dried in vacuo and characterized via NMR prior to recrystallization.

3.3.1 With *N*-methyl-*N*-phenylbenzamide (1a) no conversion was detected even after heating to 50 °C for 18 hours. See Section 5 for follow-up crossover experiments.

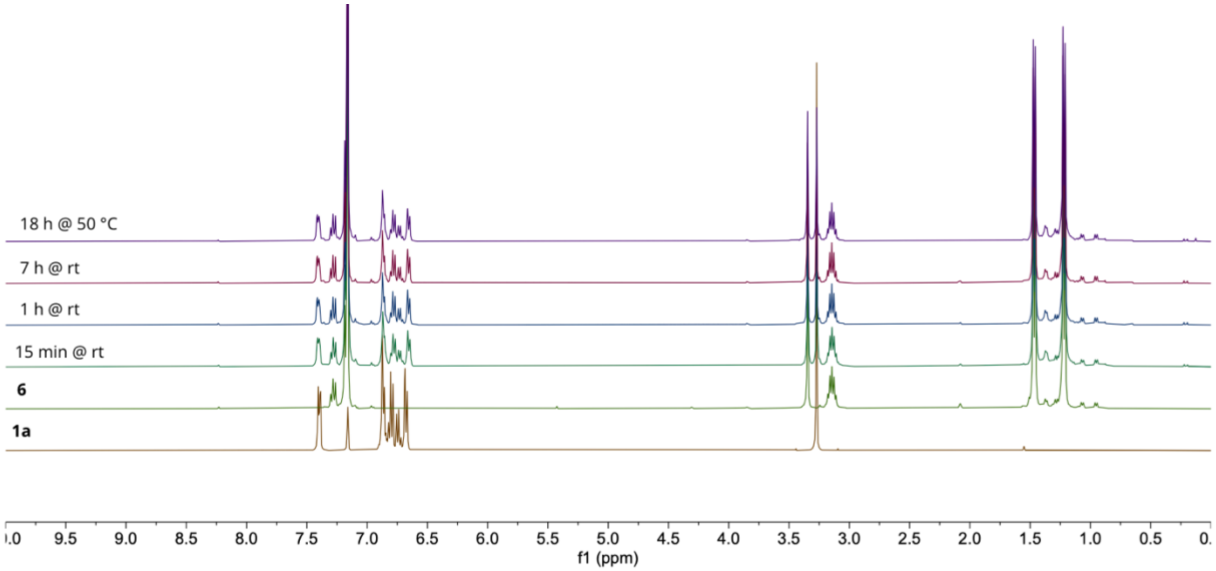

Figure S10. ^{1}H NMR (400 MHz, C_6D_6) spectra monitoring reactivity between **1a** and **6**.

3.3.2 With *N*-methyl-*N*-phenylpicolinamide (1h) negligible conversion was observed after 3 hours at ambient temperature. Upon heating to 50 °C for 21 hours, increased levels of a new product were detected. However, attempts to isolate and characterize the product(s) fully were unsuccessful.

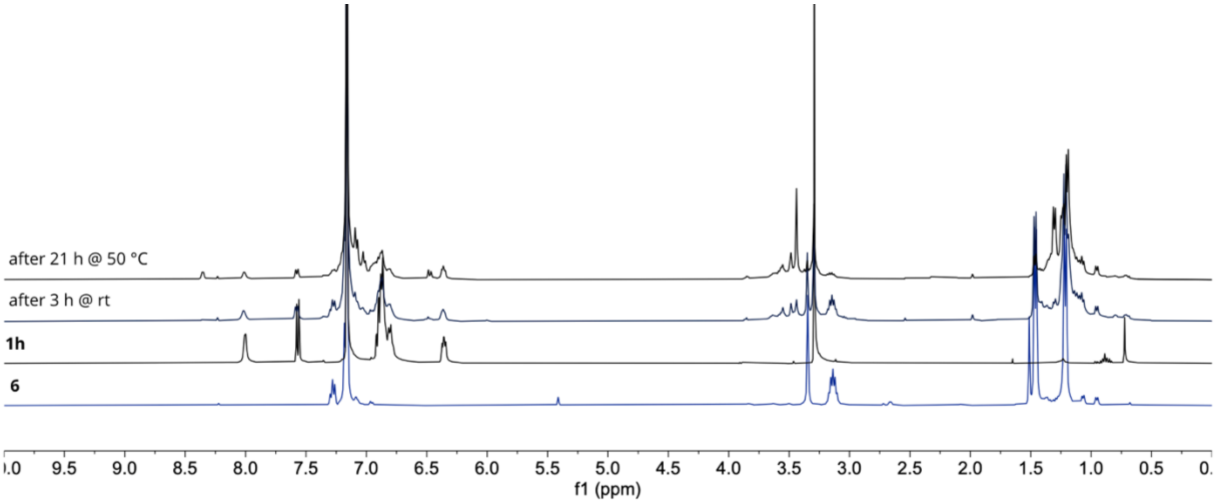

Figure S11. ^{1}H NMR (400 MHz, C_6D_6) spectra monitoring reactivity between **1h** and **6**.

3.3.3 With *N*-methyl-*N*-(pyridin-2-yl)benzamide (1i) near-complete consumption of **6** was observed within 3 hours at ambient temperature; however, remaining amide **1i** was also observed. Single-crystals suitable for X-ray diffraction analysis were obtained from the crude material and revealed to formation of oxidative addition complex **8i** (see Section 8.5). However, attempts to isolate analytically pure material have not been successful.

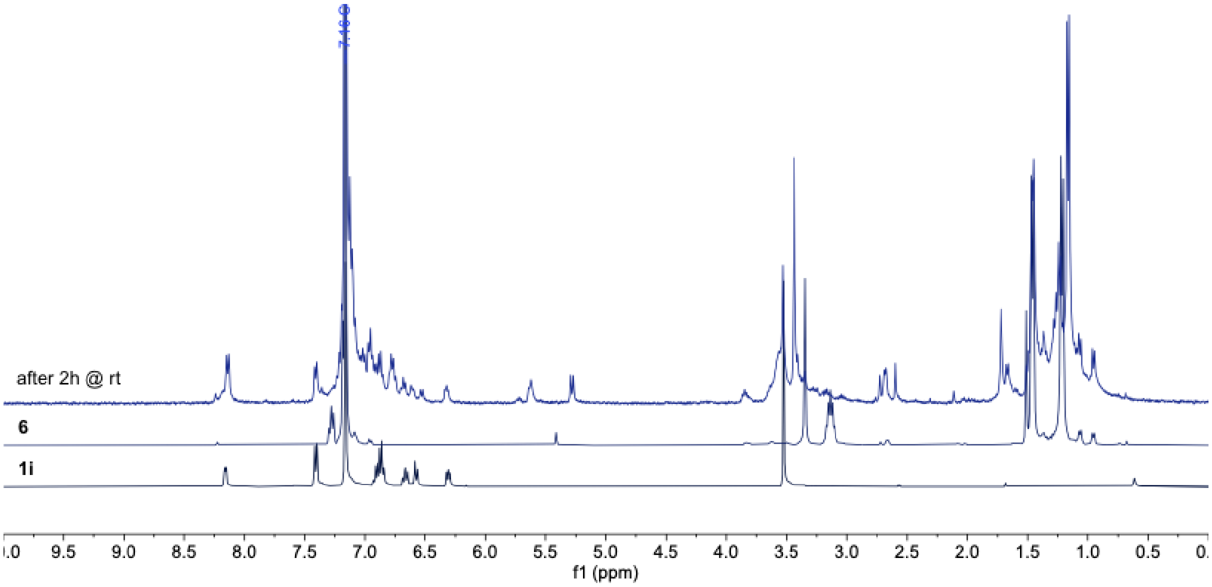

Figure S12. ^{1}H NMR (400 MHz, C_6D_6) spectra monitoring reactivity between **1i** and **6**.

4. Catalytic Reactions

4.1 Catalytic Reactions with In Situ Precatalyst Generation

Catalytic reactions were performed in analogy to the procedure reported originally.[14]

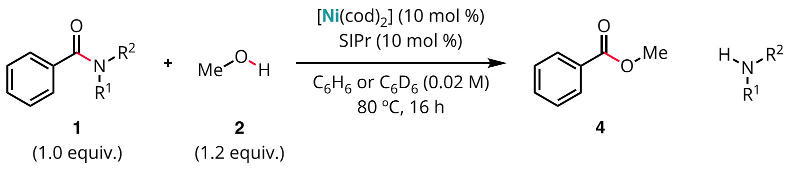

In an N_2-filled glovebox, a 1-dram vial was charged with [Ni(cod)$_2$] (10 mol %), SIPr (10 mol %), amide (0.1 mmol ,1.0 equiv.), and a magnetic stir bar. C_6H_6 or C_6D_6 (0.**4** mL) was added, and the mixture was stirred. A 0.1 mL aliquot of MeOH stock solution (1.2 M) in C_6H_6 or C_6D_6 was added, and the mixture was stirred to dissolve evenly (V_{tot} = 0.5 mL, 0.2 M). At this point, the mixture was either (i) transferred to a JYoung NMR tube or (ii) the vial was then sealed with a PTFE-lined screw cap and removed from the glovebox. The reaction mixture was stirred and heated at 80 °C in a bead bath (JYoung NMR tube) or a sand-filled heating block on a heating stir plate (vial) for 16 hours. Upon cooling to room temperature, the vial was opened to expose the catalyst to air. The reaction mixture was diluted with DCM (1 mL) and filtered through a silica plug with additional DCM (12–15 mL) and concentrated in vacuo. For ^{1}H NMR analysis, CH_2Br_2 was used as an internal standard to calculate conversion and yields. Trifluorotoluene was used as an internal standard for ^{19}F{^{1}H} NMR when applicable. Representative results are summarized below

Table S1. Representative results obtained for esterification of twisted amides using in situ catalyst generation from [Ni(cod)$_2$] and SIPr under standard conditions.

Entry	Substrate	R^1	R^2	Conversion **1** (%)	Yield **4** (%)
1	**1a**	Me	Ph	>99	81
2	**1b**	Me	Boc	--	--
3	**1c**	Ph	Boc	>99	95
4	**1d**	Boc	Boc	83	76

4.2 Catalytic Reactions with Single-Component (Pre-formed) Precatalysts

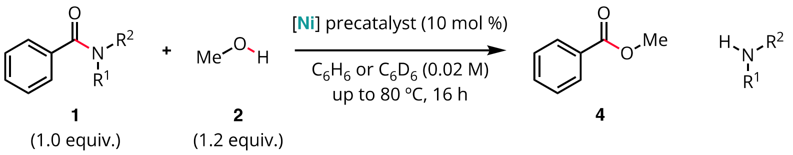

4.2.1 General Procedure: In an N_2-filled glovebox, a 1-dram vial was charged with Ni precatalyst **6**, **7**, or **8** (10 mol %), amide (0.1 mmol, 1.0 equiv.) and a magnetic stir bar. C_6H_6 or C_6D_6 (1 mL, 0.2 M) and MeOH (0.12 mmol, 1.2 equiv.) were added via syringe. At this point, the mixture was either (i) transferred to a JYoung NMR tube or (ii) the vial was then sealed with a PTFE-lined

screw cap and removed from the glovebox. The reaction mixture was heated to the indicated temperature in a bead bath (JYoung NMR tube) or a sand-filled heating block on a heating stir plate (vial) for 16 hours. Upon cooling to room temperature, the vial was opened to expose the catalyst to air. The reaction mixture was diluted with DCM (1 mL) and filtered through a silica plug with additional DCM (10–12 mL) to afford crude material for analysis. For ^{1}H NMR analysis, CH_2Br_2 was used as an internal standard to calculate conversion and yields. Trifluorotoluene was used as an internal standard for $^{19}F\{^{1}H\}$ NMR when applicable. Representative results obtained using single-component precatalyst **6** are summarized below.

Table S2. Representative results obtained for esterification of twisted amides using single-component precatalyst **6**, **7**, or **8** under standard conditions.

Entry	[Ni] Precatalyst	Substrate	R^1	R^2	Conversion **1** (%)	Yield **4** (%)
1	**6**	**1a**	Me	Ph	>99	90
2	**6**	**1b**	Me	Boc	>99	94
3	**6**	**1c**	Ph	Boc	>99	69
4	**6**	**1d**	Boc	Boc	96	72
5	**7**	**1a**	Me	Ph	86	22
6	**8c**	**1c**	Ph	Boc	99	90

4.2.2 Tests for Catalytic Competence of Ni(I): In a nitrogen-filled glovebox, a 1-dram vial was charged with Ni(I) (~10 mol%, 0.013 mmol), amide **1a** or **1c** (1.0 equiv., 0.13 mmol), and a PTFE-coated magnetic stir bar. Toluene or toluene-d_8 (0.7mL) was added using a glass microliter syringe, and the reagents were stirred to dissolve. Methanol (1.2 equiv., 0.15 mmol) was then added to this solution using a glass microliter syringe. The vial was sealed, removed from the glovebox, and maintained at 80 °C in a heated aluminum block while stirring. After 16 hours, the reaction mixture was filtered through a short silica plug using additional toluene or toluene-d_8 (~0.3mL). 1,3,5 trimethoxy benzene (1 equiv., 0.13 mmol) was added as an internal standard, and the product mixture was analyzed by both ^{1}H NMR and GC-MS.

With amide **1a**: Methyl benzoate (**4**) was generated in 22% yield upon use of $[(SIPr)Ni(OPh)]_2$ (**7**) as a precatalyst. ***However***, no new products were detected upon use of Ni(I) generated from comproportionation of **6** and **8c** as a precatalyst.

With amide **1c**: Methyl benzoate (**4**) was generated in 26% yield upon use of Ni(I) (generated from comproportionation of **6** and **8c**) as a precatalyst.

5. Crossover Experiment

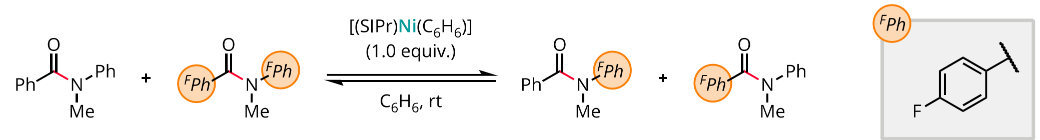

[**1a**]$_0$ = 0.10 M [**1e**]$_0$ = 0.10 M [**1f**]$_0$ = 0 M [**1g**]$_0$ = 0 M

In a nitrogen-filled glovebox, a 1-dram vial was charged with [(SIPr)Ni(C_6H_6)] (1.0 equiv., 0.04 mmol), amide **1a** (1.0 equiv, 0.04 mmol), double fluorine-labelled amide **1e** (1.0 equiv, 0.04 mmol), and a magnetic stir bar. C_6H_6 (3 mL) was added, and the reaction mixture was stirred at room temperature. After 3.5 hours, a small aliquot was removed for GCMS analysis, and the mixture was concentrated in vacuo. A GCMS sample was prepared in Et_2O. Experimental ratios of amide starting materials and crossover products were determined via GCMS.

Table S3. Diagnostic peaks detected by GC-MS support crossover.

Peak	R_t (min)	Area	Ratios	Fragment *m/z*	Identity
1	8.367	292580	1.61	123	**1e**
2	8.406	181292	1.00	123	**1g**
3	8.457	184132	1.02	105	**1f**
4	8.510	308308	1.70	105	**1a**

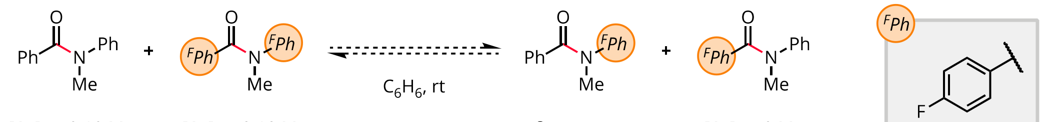

[**1a**]$_0$ = 0.10 M [**1e**]$_0$ = 0.10 M [**1f**]$_0$ = 0 M [**1g**]$_0$ = 0 M

A control experiment was conducted using the same method as described above but done in the absence of [Ni] precatalyst. In an N_2-filled glovebox, a 1-dram vial was charged with amides **1a** (1.0 equiv., 0.1 mmol) and **1e** (1.0 equiv., 0.1 mmol), and a magnetic stir bar. C_6H_6 (3 mL, 0.03 M) was added to the vial, which was stirred at room temperature inside the glovebox for 3.5 hours. Upon completion, the vial was removed from the box and an aliquot was removed for GCMS sample preparation in Et_2O. Experimental ratios of amide starting materials were determined via GCMS. No crossover products were detected.

Table S4. Diagnostic peaks detected by GC-MS indicate no background crossover.

Peak	R_t (min)	Area	Ratios	Fragment *m/z*	Identity
1	8.368	11361138	1.17	123	**1e**
2	8.509	9673816	1.00	105	**1a**

6. Comproportionation Experiments

The viability of comproportionation between Ni(0) source **6** and Ni(II) source **8c** was assessed under various conditions to obtain a qualitative assessment of its relevance to catalysis. No reaction was observed at room temperature (~22 °C) over the course of several hours.

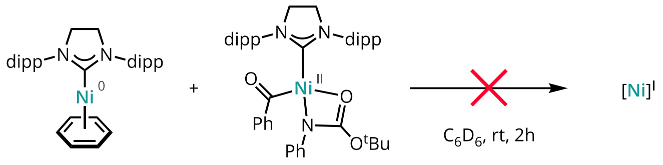

6 **8c**

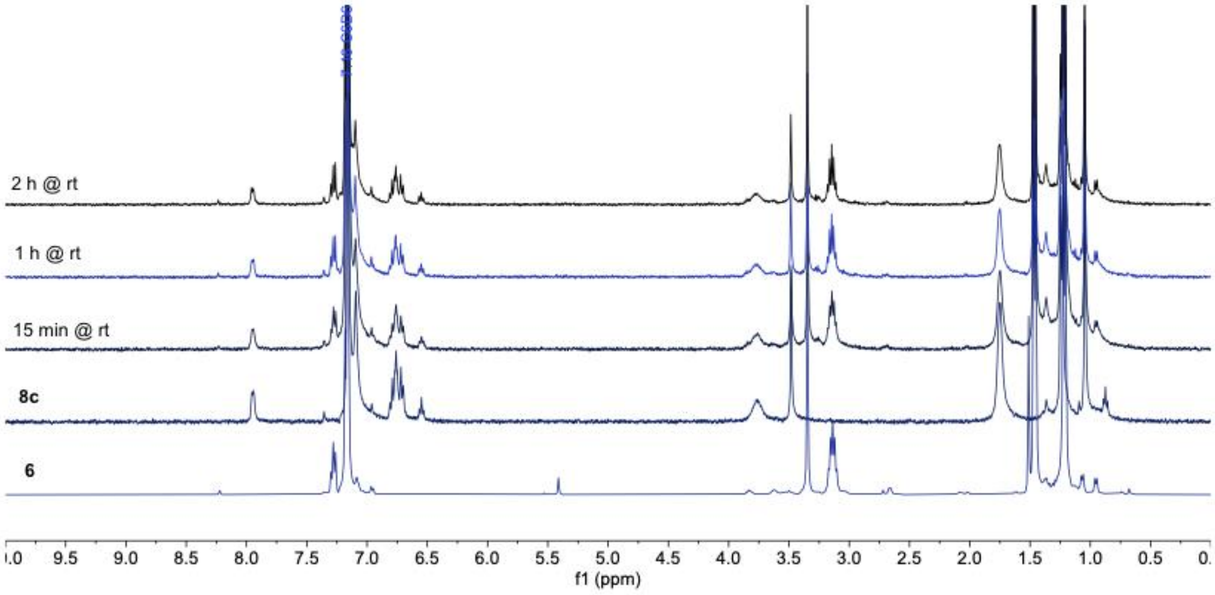

Figure S13. ^{1}H NMR (400 MHz, C_6D_6) spectra monitoring for reactivity between **6** and **8c** at rt.

Upon heating to 80 °C (the standard temperature for catalytic conditions), gradual conversion to Ni(I) was observed over several hours. As such, Ni(I) species are likely not present at early stages of catalytic reactions but may accumulate at longer reaction times, resulting in a gradual catalyst deactivation process and degraded chemoselectivity (especially for slow-reacting substrates).

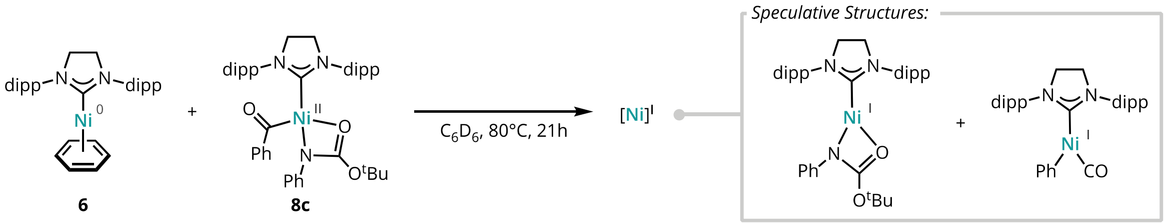

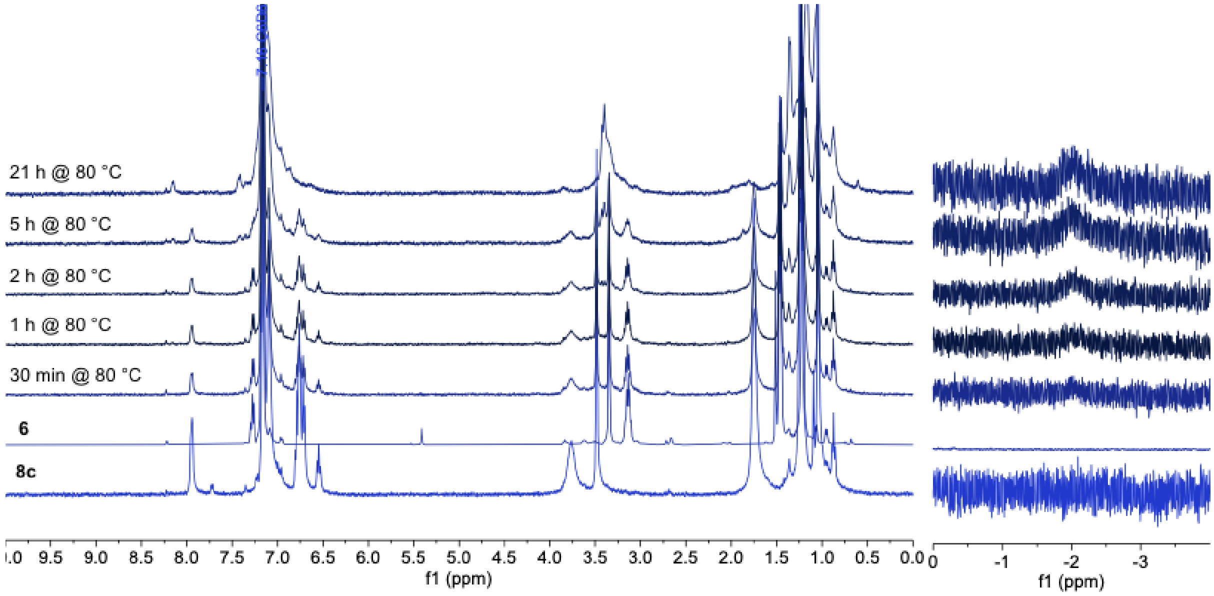

Figure S14. ^{1}H NMR (400 MHz, C_6D_6) spectra monitoring for reactivity between **6** and **8c** at 80 °C.

Diagnostic features observed using ^{1}H NMR and EPR are highlighted in Figures S15 and S16.

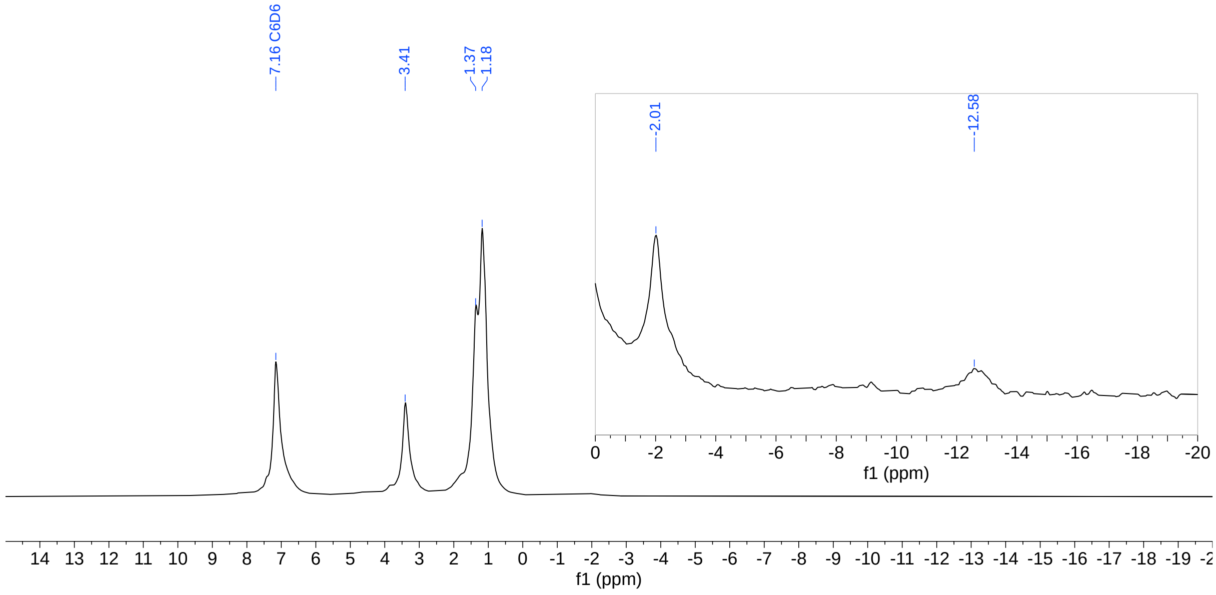

Figure S15. ^{1}H NMR (400 MHz, C_6D_6) spectrum of isolated species generated from the comproportionation of **6** and **8c.**

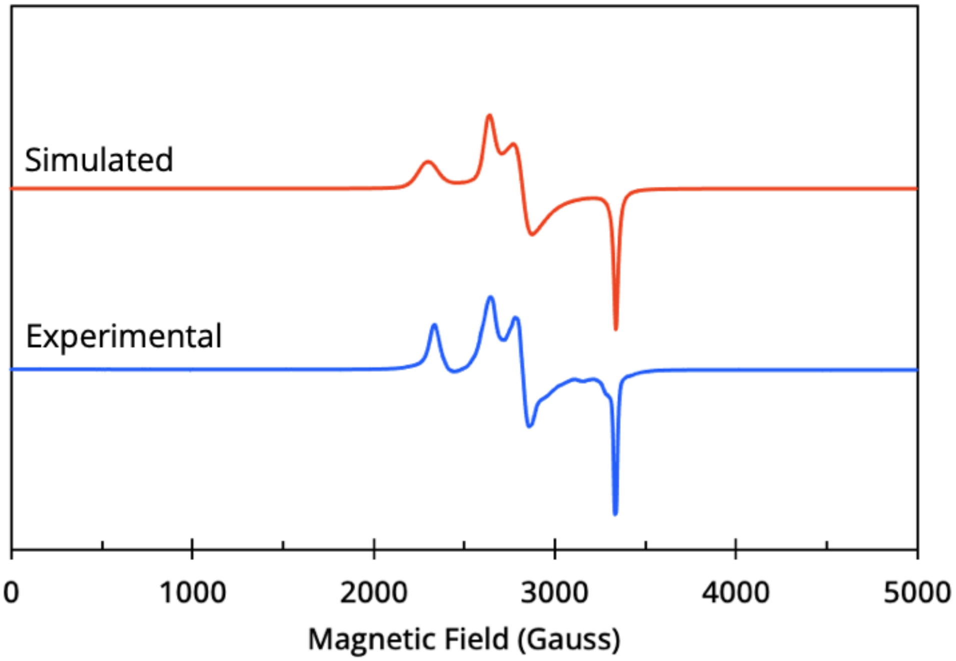

Figure S16. X-band EPR (THF glass, 10 K) spectrum of species generated from the comproportionation of **6** and **8c.**

7. Reaction Time-course Experiments

7.1 General Procedures: In a nitrogen-filled glovebox, a 1-dram vial was charged with Ni(cod)$_2$ (10 mol %, 0.01 mmol), SIPr (10 mol %, 0.01 mmol), and a magnetic stir bar. Toluene-d_8 (0.3 mL) was added to the 1-dram vial, which was stirred for 30 minutes at room temperature. Amide **1a** (1.0 equiv., 0.1 mmol) was weighed into a GC vial, and 1,3,5-trimethoxybenzene internal standard (1.0 equiv., 0.1 mmol) was weighed into a separate GC vial. After 30 minutes, the internal standard was added as a solid to the 1-dram vial containing the precatalyst mixture. A portion of toluene-d_8 (0.2 mL) was added to the GC vial to rinse out additional solid and was decanted into the 1-dram vial. The same addition procedure was repeated for amide **1a**. The reaction mixture was stirred for a minute at room temperature, then transferred to a JYoung NMR tube using a pipet. Additional toluene-d$_8$ (0.3 mL such that V_{tot} = 1.0 mL, 1.0 M) was used to effect a quantitative transfer. The sample was then sealed under N_2, removed from the glovebox, and loaded into a Bruker 500 MHz NMR spectrometer. A pseudo 2D mode was used to collect 1H NMR spectra at 5-minute intervals using the parameters described in Table X. After the first scan, the sample was replaced by a dummy JYoung NMR tube with toluene-d_8 (1 mL). The sample was pumped back into the glovebox, after which methanol (1.2 equiv., 0.12 mmol) was added directly to the NMR tube, which was sealed again under N_2. The JYoung NMR tube was removed from the glovebox, after which it was inverted, then frozen in a liquid N_2 bath to arrest the reaction during transport. The sample was then thawed, inverted to mix, and loaded into the NMR spectrometer. The sample was run for the remainder of the pseudo 2D experiment overnight (8 hours total). The next day, the sample was removed, characterized by ^{1}H NMR, then opened to air. The contents of the JYoung NMR tube were decanted into a 1-dram vial, to which CH_2Br_2 internal standard (1.0 equiv., 0.1 mmol) was added. The vial was shaken to mix, then filtered through a silica plug into an NMR tube and analyzed by ^{1}H NMR to obtain analytical yields of methyl benzoate (**4**).

Table S5. Parameters for monitoring reactions using ^{1}H NMR

Method	No. of FIDs	No. of Scans/FID	D20 (Delay)	Temperature (°C)
Pseudo 2D ^{1}H	96	1	300 sec	40

Note: The D20 value indicates the amount of time delay between collecting spectra.

Alternatively, in a nitrogen-filled glovebox, two separate 1-dram vials were charged with (A) [(SIPr)Ni(toluene)] (10 mol %, 0.009 mmol) and (B) amide **1a** (1.0 equiv., 0.09 mmol). The [Ni] complex from vial A was transferred to vial B as using small portions of toluene-d_8 (0.7 mL total). Methanol (1.2 equiv., 0.11 mmol) was added to this solution using a microliter glass syringe. All contents of vial B were transferred to a JYoung NMR tube using a glass pipet, which was then sealed and removed from the glovebox. The JYoung NMR tube was kept at 80°C in a bead bath, and ^{1}H NMR spectra was collected at 25 °C at the indicated time intervals. Conversion was monitored through the relative integrals of diagnostic signals for amide **1a** and ester **4**. After 21h, the reaction mixture was filtered through a silica plug and 1,3,5 trimethoxy benzene internal standard (1 equiv., 0.09 mmol) was added as an internal standard, and a final 1H NMR spectrum was collected to obtain analytical yields of methyl benzoate (**4**), which were within 2–4% of the values expected from ratios obtained during the reaction time-course.

7.2 Reaction Time-course at 80 °C: Initial reaction time-course experiments were conducted at 80°C in toluene-d_8 to most closely resemble standard catalytic conditions. Initial rates were too fast to collect data at low conversion. Nonetheless, three key observations were noted.

(1) Using single-component [(SIPr)Ni(arene)] resulted in markedly faster reaction rates compared with the corresponding in situ precatalyst activation protocol. See Figure S17.

(2) Using the in situ precatalyst activation protocol in toluene-d_8 resulted in precatalyst speciation challenges analogous to those noted in benzene-d_6. Namely, [Ni(cod)$_2$] persisted as a major component of the Ni-containing species throughout the course of the reaction rather than accessing the active SIPr-supported form. See Figure S20.

(3) Using the pre-formed precatalyst protocol, [(SIPr)Ni(arene)] remained the predominant Ni-containing species detected at high conversion. See Figure S21.

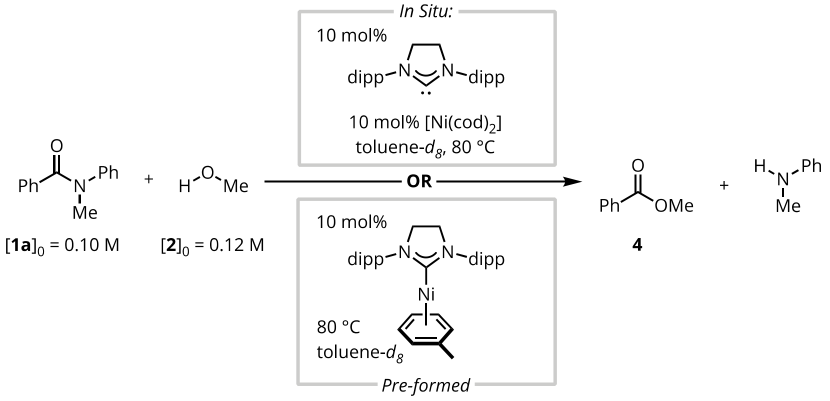

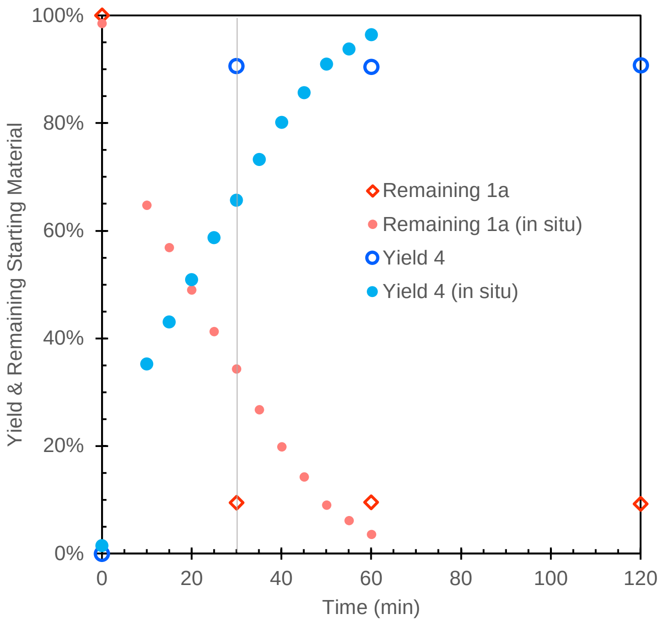

Figure S17. Reaction time-course obtained upon monitoring with ^{1}H NMR where [**1a**]$_0$ = 0.1 M, [**2**]$_0$ = 1.2 M, and [(SIPr)Ni(toluene)]$_0$ (*pre-formed*) or [SIPr]/[Ni(cod)$_2$] (*in situ*) = 0.01 M at 80 °C.

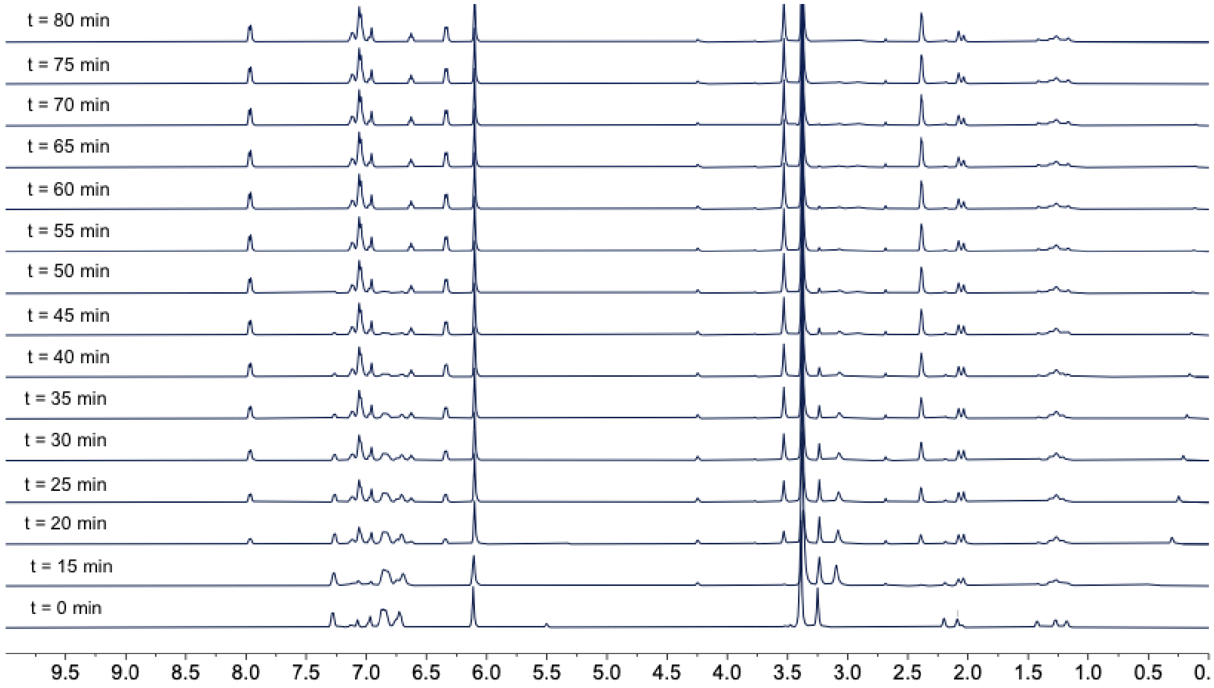

Figure S18. ^{1}H NMR spectra collected at t = 0 min (bottom) through 800 min (top) where [**1a**]$_0$ = 0.1 M, [**2**]$_0$ = 1.2 M, [SIPr]$_0$ = 0.01 M, and [Ni(cod)$_2$]$_0$ = 0.01 M at 80 °C (*in situ*).

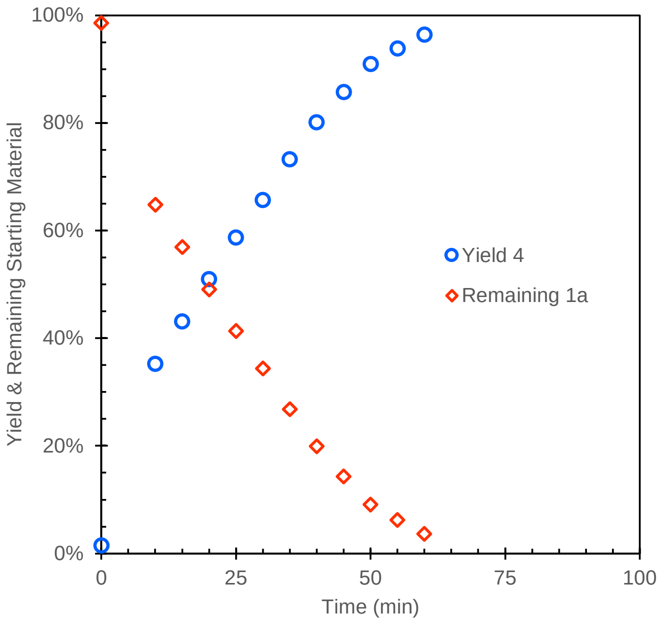

Figure S19. Reaction time-course obtained upon monitoring with ^{1}H NMR where [**1a**]$_0$ = 0.1 M, [**2**]$_0$ = 1.2 M, [SIPr]$_0$ = 0.01 M, and [Ni(cod)$_2$]$_0$ = 0.01 M at 80 °C (*in situ*).

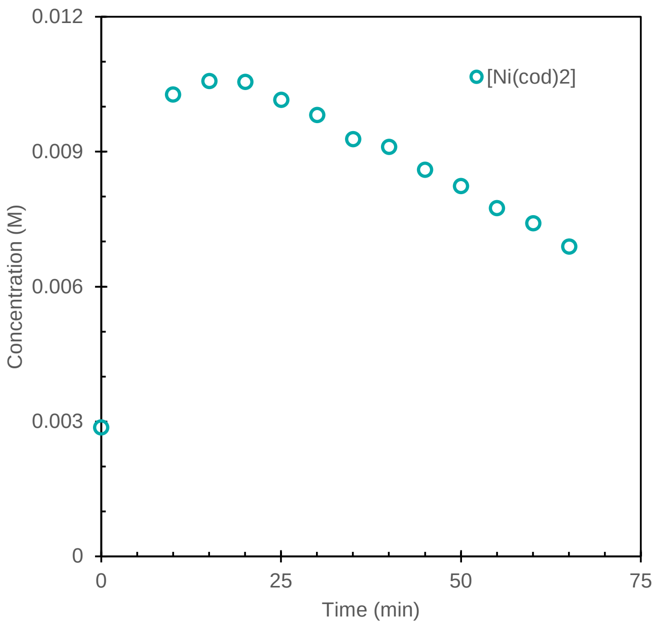

Figure S20. Concentration of [Ni(cod)$_2$] remaining over the reaction time-course obtained upon monitoring by ^{1}H NMR where [**1a**]$_0$ = 0.1 M, [**2**]$_0$ = 1.2 M, [SIPr]$_0$ = 0.01 M, and [Ni(cod)$_2$]$_0$ = 0.01 M at 80 °C (*in situ*).

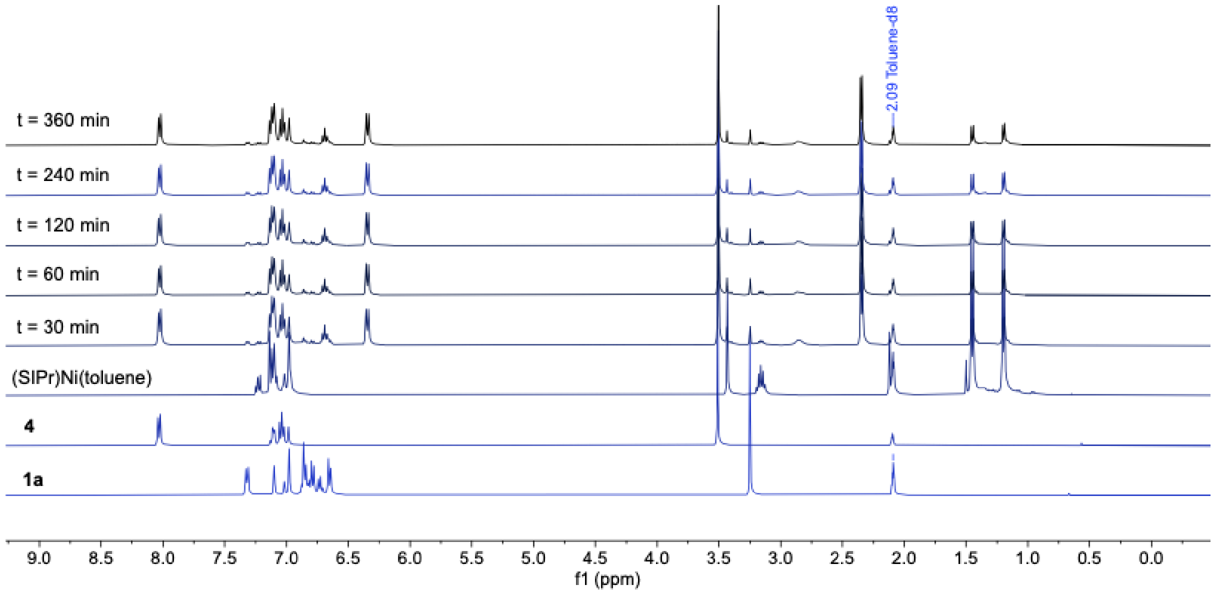

Figure S21. ^{1}H NMR spectra collected where [**1a**]$_0$ = 0.1 M, [**2**]$_0$ = 1.2 M, and [(SIPr)Ni(toluene)]$_0$ = 0.01 M kept at 80 °C between timepoints (*pre-formed*).

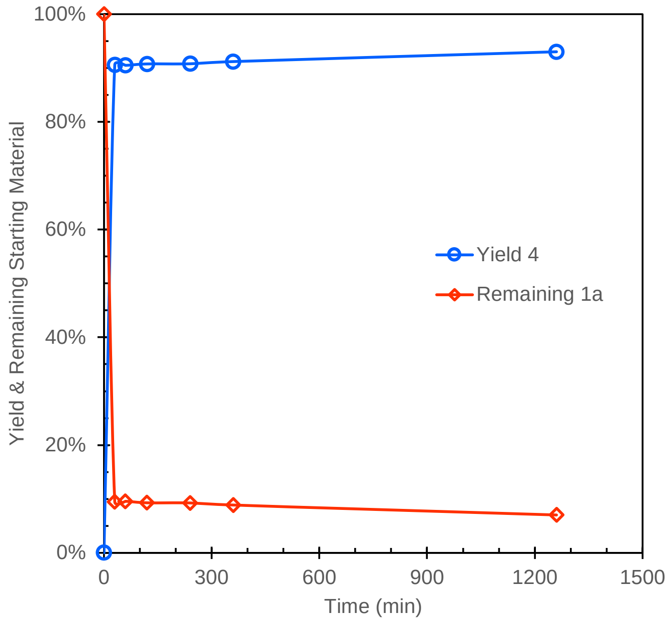

Figure S22. Reaction time-course obtained upon monitoring with ^{1}H NMR where [**1a**]$_0$ = 0.1 M, [**2**]$_0$ = 1.2 M, and [(SIPr)Ni(toluene)]$_0$ = 0.01 M kept at 80 °C between timepoints *(pre-formed)*.

7.3 Reaction Time-course at 40 °C: To facilitate data collection at low conversion, subsequent reaction time-course experiments were conducted at 40 °C in toluene-d_8. The lower reaction temperature facilitated head-to-head comparison of in situ precatalyst formation and the single-component (pre-formed) precatalyst. These experiments validated that single-component [(SIPr)Ni(arene)] resulted in markedly faster reaction rates compared with the corresponding in situ precatalyst activation protocol. However, conversion stalled at ~60% yield **4**, suggesting competitive catalyst deactivation with a high kinetic barrier preventing re-entering to the catalytic cycle at 40 °C.

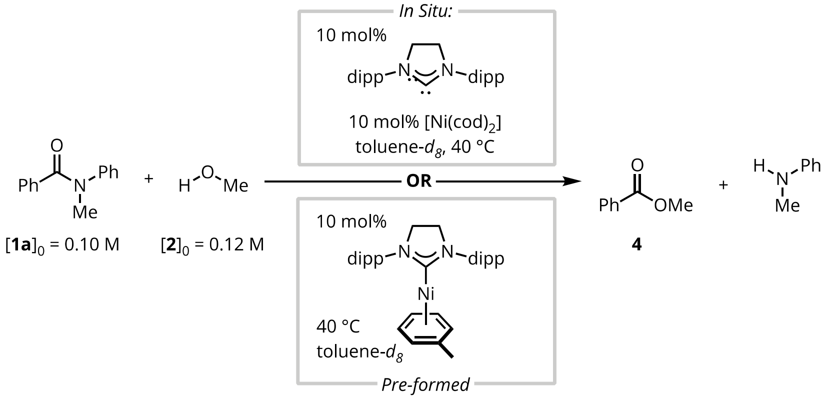

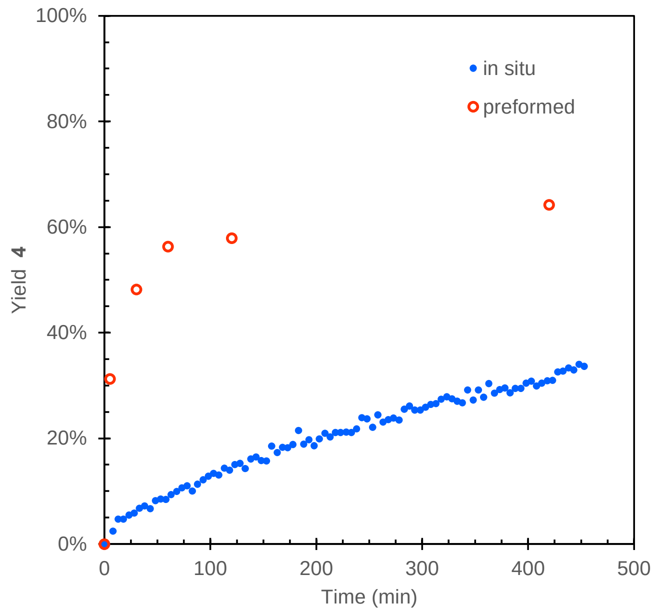

Figure S23. Reaction time-course obtained upon monitoring with ^{1}H NMR where [**1a**]$_0$ = 0.1 M, [**2**]$_0$ = 1.2 M, and [(SIPr)Ni(toluene)]$_0$ (*pre-formed*) or [SIPr]/[Ni(cod)$_2$] (*in situ*) = 0.01 M at 40 °C.

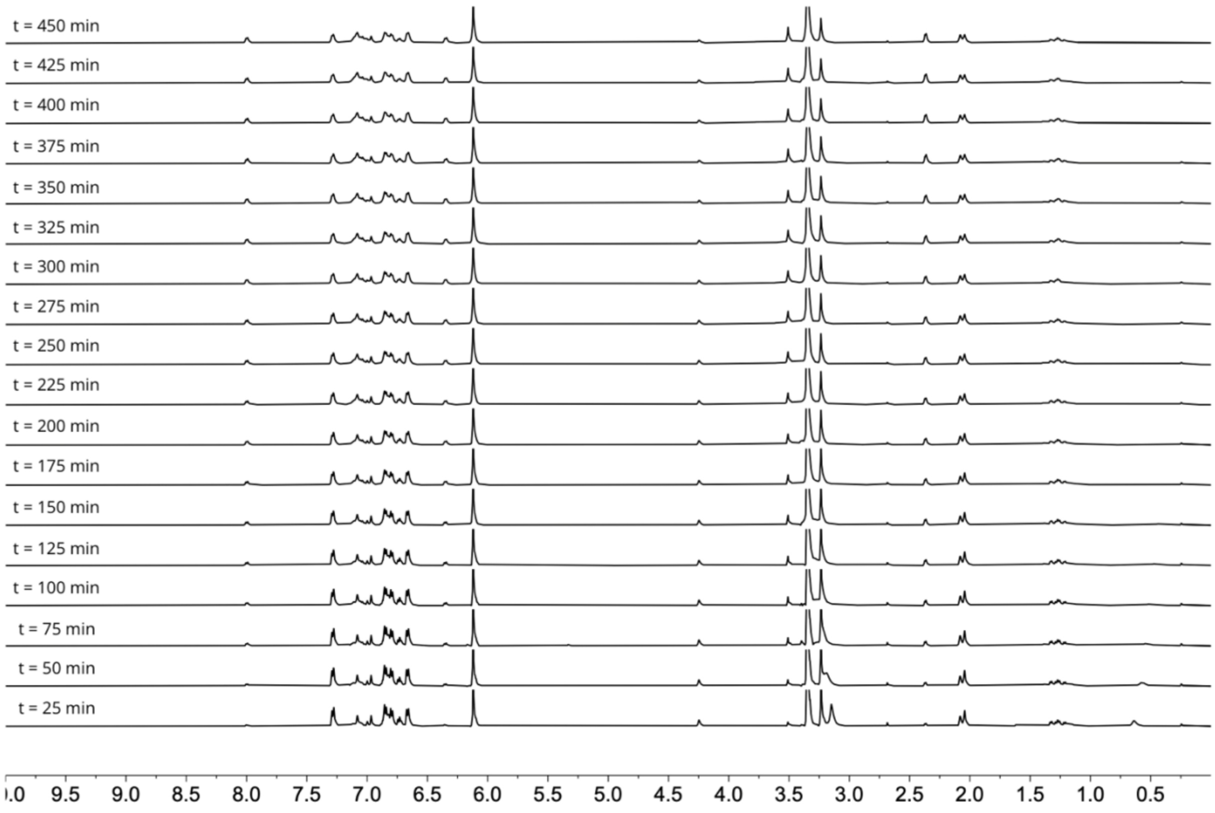

Figure S24. ^{1}H NMR spectra collected at varied time (t) where [**1a**]$_0$ = 0.1 M, [**2**]$_0$ = 1.2 M, [SIPr]$_0$ = 0.01 M, and [Ni(cod)$_2$]$_0$ = 0.01 M at 40 °C (*in situ*).

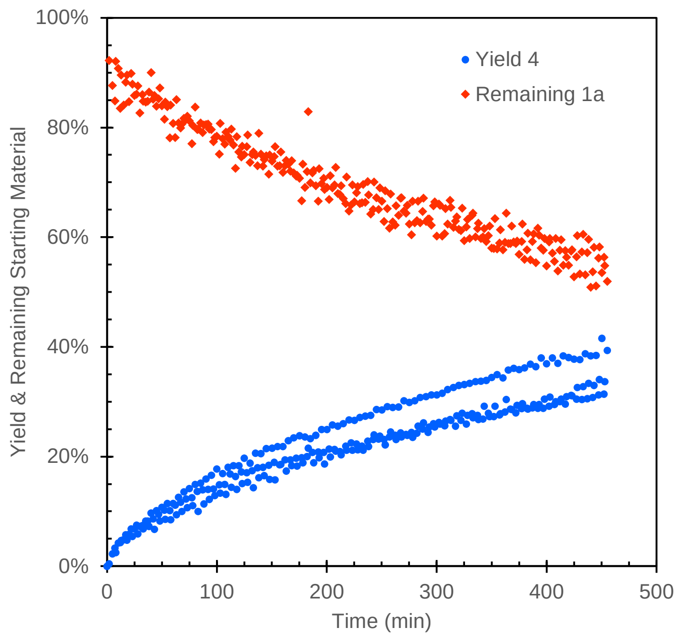

Figure S25. Reaction time-course obtained upon monitoring with ^{1}H NMR where [**1a**]$_0$ = 0.1 M, [**2**]$_0$ = 1.2 M, [SIPr]$_0$ = 0.01 M, and [Ni(cod)$_2$]$_0$ = 0.01 M at 40 °C (*in situ*).

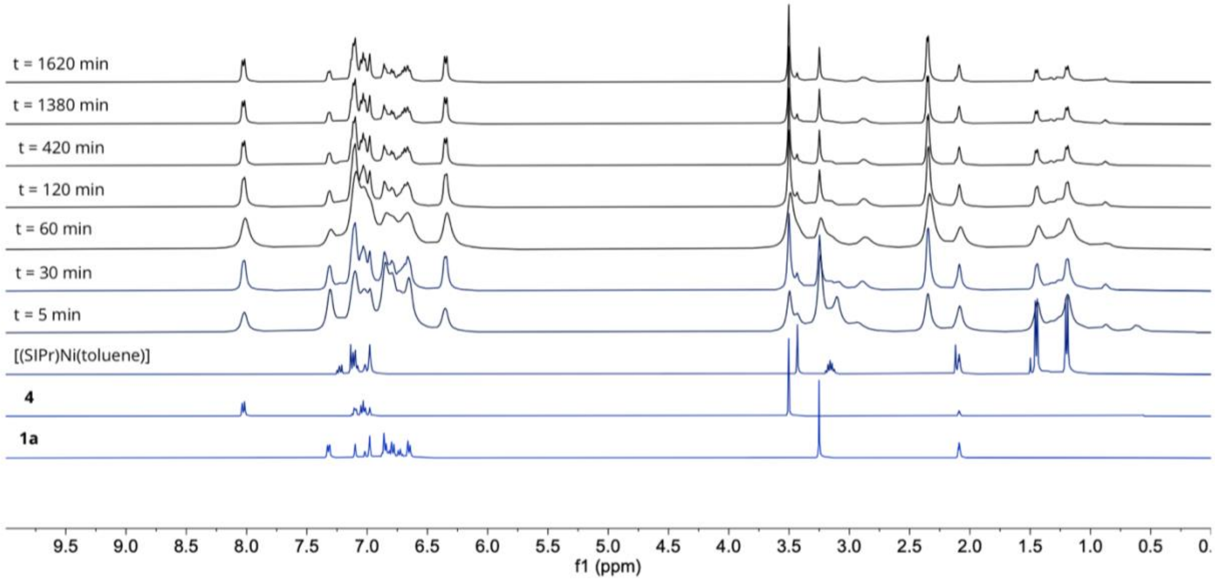

Figure S26. ^{1}H NMR spectra collected where [**1a**]$_0$ = 0.1 M, [**2**]$_0$ = 1.2 M, and [(SIPr)Ni(toluene)]$_0$ = 0.01 M kept at 40 °C between timepoints (*pre-formed*).

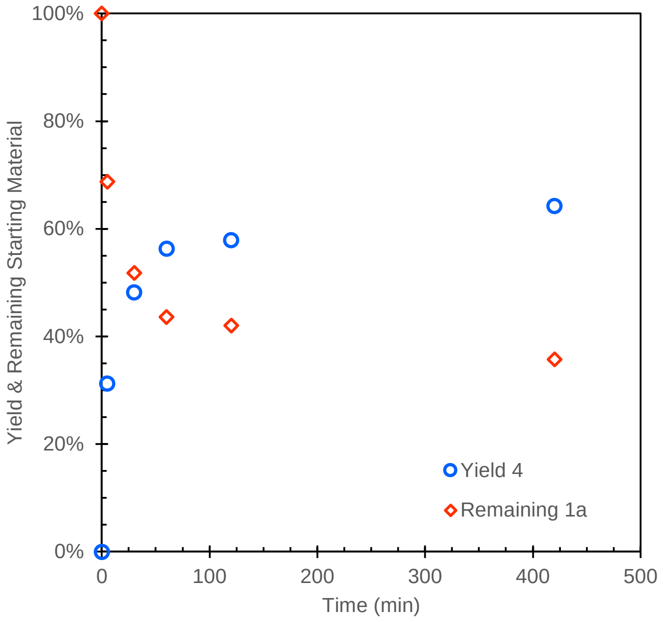

Figure S27. Reaction time-course obtained upon monitoring with ^{1}H NMR where [**1a**]$_0$ = 0.1 M, [**2**]$_0$ = 1.2 M, and [(SIPr)Ni(toluene)]$_0$ = 0.01 M kept at 40 °C between timepoints *(pre-formed)*.

8. Single-Crystal X-Ray Diffraction Data

8.1 General Procedure: A single crystal was placed onto a thin glass optical fiber or a nylon loop and mounted on a Rigaku XtaLAB Synergy-S Dualflex diffractometer equipped with a HyPix-6000HE HPC area detector for data collection at 100.00(10) K. A preliminary set of cell constants and an orientation matrix were calculated from a small sampling of reflections.[15] short pre-experiment was run, from which an optimal data collection strategy was determined. The full data collection was carried out using a PhotonJet (Cu) X-ray source with a detector distance of 34.0 mm. Series of frames were collected in 0.50° steps in ω at different 2θ, κ, and φ settings. After the intensity data were corrected for absorption, the final cell constants were calculated from the xyz centroids of strong reflections from the actual data collection after integration.[15] Structures were solved using SHELXT2[16] and refined using SHELXL.[17] The space group was determined based on systematic absences and intensity statistics. Most or all non-hydrogen atoms were assigned from the solution. Full-matrix least squares / difference Fourier cycles were performed which located any remaining non-hydrogen atoms. All non-hydrogen atoms were refined with anisotropic displacement parameters.

Data collection, structure solution, and structure refinement were conducted at the X-ray Crystallographic Facility, B04 Hutchison Hall, Department of Chemistry, University of Rochester. The instrument was purchased with funding from NSF MRI program grant CHE-1725028.

8.2 Solid-State Structure of 8b *(CCDC 2264311)*

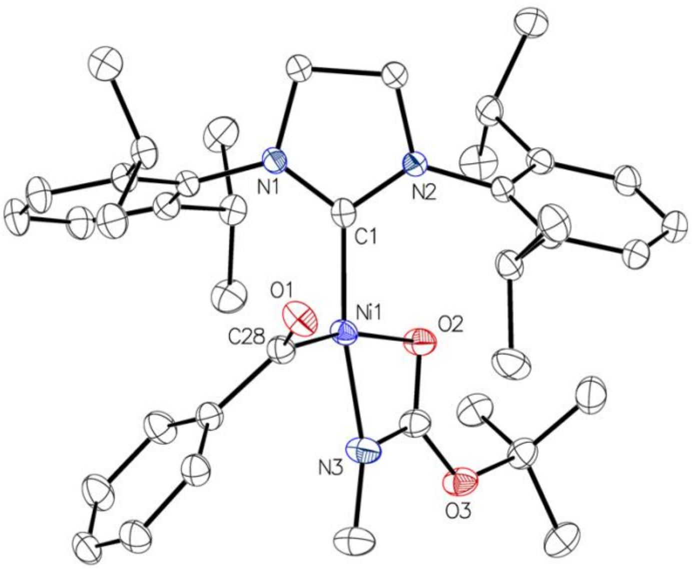

Figure S28. Solid-state structure of **8b** determined by SC-XRD. Anisotropic displacement ellipsoids drawn at the 50 % probability level. H atoms omitted for clarity. Figure generated with OLEX2.[18]

Table S6. Crystal data and structure refinement for **8b**.

Empirical formula	C40 H55 N3 Ni O3	
Formula weight	684.58	
Temperature	100.01(10) K	
Wavelength	1.54184 Å	
Crystal system	triclinic	
Space group	*P*-1	
Unit cell dimensions	*a* = 11.45670(10) Å	a = 77.0930(10)°
	b = 12.4682(2) Å	b = 83.9970(10)°
	c = 13.81530(10) Å	g = 88.7420(10)°
Volume	1913.03(4) Å^3	
Z	2	
Density (calculated)	1.188 Mg/m^3	
Absorption coefficient	1.027 mm^{-1}	
F(000)	736	
Crystal color, morphology	yellow, plate	
Crystal size	0.121 x 0.07 x 0.033 mm^3	
Theta range for data collection	3.299 to 80.373°	
Index ranges	$-14 < h < 14$, $-13 < k < 15$, $-17 < l < 17$	
Reflections collected	64991	
Independent reflections	8230 [*R*(int) = 0.0383]	
Observed reflections	7513	
Completeness to theta = 74.504°	99.7%	
Absorption correction	Multi-scan	
Max. and min. transmission	1.00000 and 0.93347	
Refinement method	Full-matrix least-squares on F^2	
Data / restraints / parameters	8230 / 0 / 436	
Goodness-of-fit on F^2	1.070	
Final *R* indices [*I*>2sigma(*I*)]	*R*1 = 0.0328, *wR*2 = 0.0801	
R indices (all data)	*R*1 = 0.0358, *wR*2 = 0.0817	
Largest diff. peak and hole	0.280 and -0.393 e.Å^{-3}	

Table S7. Atomic coordinates (x 10^4) and equivalent isotropic displacement parameters (Å^{2}x 10^3) for **8b**. U_{eq} is defined as one third of the trace of the orthogonalized U_{ij} tensor.

	x	y	z	U_{eq}
Ni1	6777(1)	6653(1)	7257(1)	17(1)
O1	7792(1)	5906(1)	5653(1)	26(1)
O2	6045(1)	7727(1)	8079(1)	23(1)
O3	4053(1)	7995(1)	7996(1)	25(1)
N1	9191(1)	6094(1)	7579(1)	16(1)
N2	8932(1)	7840(1)	6993(1)	16(1)
N3	5091(1)	6706(1)	7310(1)	24(1)
C1	8378(1)	6865(1)	7295(1)	16(1)
C2	10352(1)	6579(1)	7590(1)	19(1)
C3	10220(1)	7729(1)	6948(1)	19(1)
C4	8933(1)	4989(1)	8120(1)	17(1)
C5	9400(1)	4123(1)	7701(1)	19(1)
C6	9213(1)	3050(1)	8258(1)	24(1)
C7	8586(1)	2842(1)	9191(1)	25(1)
C8	8128(1)	3708(1)	9591(1)	22(1)
C9	8293(1)	4797(1)	9072(1)	18(1)
C10	10110(1)	4302(1)	6684(1)	20(1)
C11	9587(1)	3676(1)	5994(1)	25(1)
C12	11395(1)	3966(1)	6795(1)	26(1)
C13	7809(1)	5715(1)	9567(1)	19(1)
C14	6500(1)	5557(1)	9928(1)	28(1)
C15	8499(1)	5800(1)	10435(1)	30(1)
C16	8399(1)	8910(1)	6759(1)	17(1)
C17	8020(1)	9318(1)	5814(1)	19(1)
C18	7567(1)	10386(1)	5617(1)	23(1)
C19	7511(1)	11025(1)	6322(1)	25(1)
C20	7895(1)	10603(1)	7250(1)	22(1)

C21	8343(1)	9539(1)	7490(1)	19(1)
C22	8091(1)	8645(1)	5020(1)	22(1)
C23	6873(1)	8460(1)	4730(1)	32(1)
C24	8910(1)	9190(1)	4102(1)	28(1)
C25	8752(1)	9096(1)	8517(1)	20(1)
C26	7734(1)	8966(1)	9341(1)	29(1)
C27	9718(1)	9819(1)	8716(1)	27(1)
C28	7110(1)	5700(1)	6410(1)	19(1)
C29	6360(1)	4676(1)	6616(1)	19(1)
C30	6024(1)	4300(1)	5809(1)	22(1)
C31	5255(1)	3418(1)	5967(1)	28(1)
C32	4847(1)	2887(1)	6931(1)	28(1)
C33	5215(1)	3232(1)	7739(1)	30(1)
C34	5956(1)	4135(1)	7579(1)	26(1)
C35	4054(1)	6378(1)	6932(1)	31(1)
C36	5076(1)	7485(1)	7804(1)	21(1)
C37	4016(1)	8918(1)	8499(1)	23(1)
C38	4301(1)	8510(1)	9571(1)	27(1)
C39	4823(1)	9842(1)	7926(1)	32(1)
C40	2737(1)	9273(1)	8461(1)	29(1)

8.3 Solid-State Structure of 8c•0.75 Et_2O *(CCDC 2264313)*

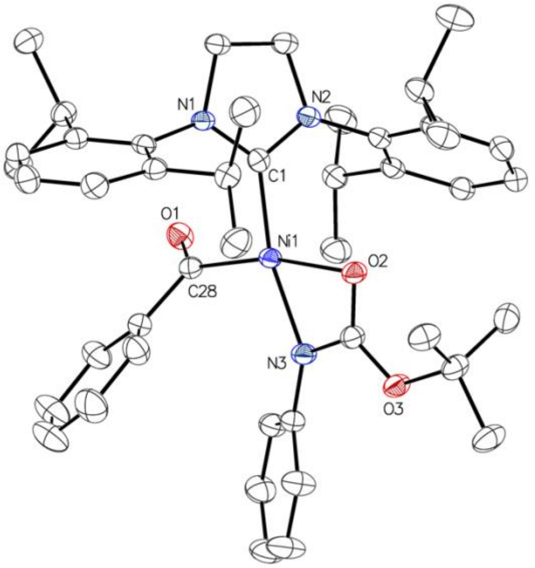

Figure S29. Solid-state structure of **8c**•0.75 Et_2O. Anisotropic displacement ellipsoids drawn at the 50 % probability level. H atoms and solvent molecules omitted for clarity. Figure generated with OLEX2[18].

Table S8. Crystal data and structure refinement for **8c**•0.75 Et_2O.

Empirical formula	C48 H64.50 N3 Ni O3.75	
Formula weight	802.23	
Temperature	100.00(10) K	
Wavelength	1.54184 Å	
Crystal system	triclinic	
Space group	*P*-1	
Unit cell dimensions	*a* = 12.41379(6) Å	a = 84.4929(5)°
	b = 16.24751(10) Å	b = 87.8704(4)°
	c = 22.60096(13) Å	g = 80.3202(5)°
Volume	4471.79(4) Å^3	
Z	4	
Density (calculated)	1.192 Mg/m^3	
Absorption coefficient	0.964 mm^{-1}	
F(000)	1726	
Crystal color, morphology	yellow, needle	
Crystal size	0.103 x 0.036 x 0.029 mm^3	
Theta range for data collection	3.248 to 80.295°	
Index ranges	-15 < *h* < 15, -20 < *k* < 20, -28 < *l* < 28	
Reflections collected	113628	
Independent reflections	19229 [*R*(int) = 0.0406]	
Observed reflections	16856	
Completeness to theta = 74.504°	99.7%	
Absorption correction	Multi-scan	
Max. and min. transmission	1.00000 and 0.84156	
Refinement method	Full-matrix least-squares on F^2	
Data / restraints / parameters	19229 / 61 / 1101	
Goodness-of-fit on F^2	1.057	
Final *R* indices [*I*>2sigma(*I*)]	*R*1 = 0.0393, *wR*2 = 0.0994	
R indices (all data)	*R*1 = 0.0456, *wR*2 = 0.1029	
Largest diff. peak and hole	0.561 and -0.499 e.Å^{-3}	

Table S9. Atomic coordinates (x 10^4) and equivalent isotropic displacement parameters (Å^{2}x 10^3) for **8c**•0.75 Et_2O. U_{eq} is defined as one third of the trace of the orthogonalized U_{ij} tensor.

Atom	x	y	z	U_{eq}
Ni1	5266(1)	1244(1)	2909(1)	18(1)
O1	6482(1)	2442(1)	2462(1)	26(1)
O2	4298(1)	509(1)	3397(1)	22(1)
O3	5172(1)	-448(1)	4111(1)	26(1)
N1	4152(1)	2142(1)	1860(1)	19(1)
N2	3488(1)	2559(1)	2703(1)	20(1)
N3	6055(1)	470(1)	3510(1)	22(1)
C1	4249(1)	2002(1)	2452(1)	18(1)
C2	3202(1)	2790(1)	1685(1)	23(1)
C3	2897(1)	3188(1)	2265(1)	25(1)
C4	4674(1)	1599(1)	1431(1)	19(1)
C5	4425(1)	784(1)	1431(1)	21(1)
C6	4886(1)	310(1)	976(1)	26(1)
C7	5563(1)	624(1)	541(1)	29(1)
C8	5806(1)	1422(1)	553(1)	25(1)
C9	5366(1)	1925(1)	999(1)	21(1)
C10	3669(1)	414(1)	1892(1)	25(1)
C11	4098(2)	-500(1)	2105(1)	37(1)
C12	2514(1)	492(1)	1656(1)	38(1)
C13	5660(1)	2796(1)	1002(1)	23(1)
C14	6899(1)	2770(1)	982(1)	28(1)
C15	5144(1)	3387(1)	479(1)	28(1)
C16	3238(1)	2543(1)	3328(1)	20(1)
C17	2339(1)	2173(1)	3543(1)	23(1)
C18	2114(1)	2143(1)	4154(1)	26(1)
C19	2743(1)	2479(1)	4530(1)	28(1)
C20	3607(1)	2860(1)	4304(1)	25(1)

C21	3875(1)	2902(1)	3699(1)	21(1)
C22	1611(1)	1824(1)	3136(1)	27(1)
C23	1532(1)	906(1)	3322(1)	33(1)
C24	470(1)	2366(1)	3104(1)	37(1)
C25	4809(1)	3342(1)	3456(1)	23(1)
C26	5836(1)	3064(1)	3825(1)	32(1)
C27	4463(1)	4295(1)	3420(1)	34(1)
C28	6408(1)	1700(1)	2525(1)	20(1)
C29	7359(1)	1064(1)	2329(1)	22(1)
C30	8386(1)	1306(1)	2267(1)	32(1)
C31	9295(1)	735(1)	2127(1)	45(1)
C32	9186(2)	-80(1)	2046(1)	50(1)
C33	8162(2)	-321(1)	2088(1)	44(1)
C34	7247(1)	253(1)	2232(1)	31(1)
C35	7093(1)	216(1)	3759(1)	22(1)
C36	7516(1)	-615(1)	3944(1)	31(1)
C37	8553(1)	-820(1)	4179(1)	40(1)
C38	9190(1)	-213(1)	4233(1)	36(1)
C39	8784(1)	611(1)	4042(1)	31(1)
C40	7748(1)	824(1)	3806(1)	26(1)
C41	5154(1)	169(1)	3675(1)	21(1)
C42	4160(1)	-758(1)	4332(1)	26(1)
C43	3660(1)	-1125(1)	3835(1)	30(1)
C44	3385(1)	-62(1)	4599(1)	31(1)
C45	4594(2)	-1445(1)	4810(1)	36(1)
Ni2	764(1)	3920(1)	8132(1)	20(1)
O4	-1053(1)	5003(1)	7724(1)	32(1)
O5	1940(1)	3239(1)	8693(1)	26(1)
O6	1318(1)	2248(1)	9355(1)	35(1)
N4	1573(1)	4863(1)	7087(1)	21(1)
N5	1969(1)	5254(1)	7934(1)	21(1)

N6	196(1)	3149(1)	8710(1)	25(1)
C46	1487(1)	4698(1)	7680(1)	20(1)
C47	2258(1)	5509(1)	6918(1)	26(1)
C48	2296(1)	5907(1)	7498(1)	26(1)
C49	1357(1)	4310(1)	6664(1)	22(1)
C50	2058(1)	3542(1)	6622(1)	26(1)
C51	1826(2)	3033(1)	6198(1)	35(1)
C52	936(2)	3274(1)	5832(1)	36(1)
C53	266(1)	4034(1)	5877(1)	30(1)
C54	469(1)	4574(1)	6287(1)	24(1)
C55	3063(1)	3260(1)	7003(1)	31(1)
C56	3039(2)	2409(1)	7352(1)	41(1)
C57	4108(2)	3241(2)	6618(1)	51(1)
C58	-226(1)	5436(1)	6295(1)	25(1)
C59	-1451(1)	5413(1)	6274(1)	34(1)
C60	108(1)	6010(1)	5767(1)	35(1)
C61	2160(1)	5229(1)	8557(1)	21(1)
C62	3207(1)	4870(1)	8758(1)	23(1)
C63	3388(1)	4827(1)	9365(1)	26(1)
C64	2568(1)	5139(1)	9757(1)	27(1)
C65	1552(1)	5511(1)	9546(1)	26(1)
C66	1325(1)	5569(1)	8941(1)	23(1)
C67	4130(1)	4533(1)	8338(1)	27(1)
C68	4489(1)	3587(1)	8475(1)	34(1)
C69	5096(1)	5008(1)	8351(1)	38(1)
C70	217(1)	6003(1)	8716(1)	25(1)
C71	-716(1)	5644(1)	9054(1)	31(1)
C72	98(1)	6951(1)	8759(1)	37(1)
C73	-533(1)	4297(1)	7734(1)	24(1)
C74	-1003(1)	3608(1)	7477(1)	29(1)
C75	-2128(2)	3684(2)	7428(1)	48(1)

C76	-2566(2)	3029(2)	7222(1)	71(1)
C77	-1898(2)	2319(2)	7060(1)	68(1)
C78	-777(2)	2244(2)	7098(1)	54(1)
C79	-338(2)	2887(1)	7315(1)	38(1)
C80	-756(1)	2802(1)	8857(1)	26(1)
C81	-780(2)	1958(1)	8866(1)	64(1)
C82	-1764(2)	1663(2)	8963(2)	81(1)
C83	-2726(2)	2200(2)	9058(1)	47(1)
C84	-2689(2)	3024(2)	9076(1)	47(1)
C85	-1714(1)	3330(1)	8973(1)	40(1)
C86	1179(1)	2873(1)	8920(1)	25(1)
C87	2387(1)	1964(1)	9631(1)	32(1)
C88	3217(2)	1601(1)	9179(1)	43(1)
C89	2744(2)	2672(1)	9919(1)	41(1)
C90	2140(2)	1284(1)	10102(1)	50(1)
C91	8480(2)	2302(2)	5165(2)	70(1)
C92	7756(2)	3005(1)	5441(1)	53(1)
O7	7518(1)	3690(1)	5006(1)	39(1)
C93	6759(2)	4352(1)	5209(1)	41(1)
C94	6637(2)	5075(1)	4741(1)	54(1)
C95	-1060(20)	928(15)	562(12)	61(7)
C96	-196(7)	154(5)	530(4)	46(2)
O8	130(20)	14(18)	-25(14)	48(4)
C97	958(9)	-478(7)	-288(5)	63(2)
C98	694(11)	-1297(8)	-436(8)	85(4)
C95'	-1350(30)	850(30)	620(20)	79(9)
C96'	-241(12)	534(11)	363(8)	55(3)
O8'	-140(20)	-216(10)	149(9)	57(3)
C97'	740(10)	-761(7)	-28(6)	45(3)
C98'	840(30)	-870(20)	-683(9)	67(6)

8.4 Solid-State Structure of 8d *(CCDC 2264312)*

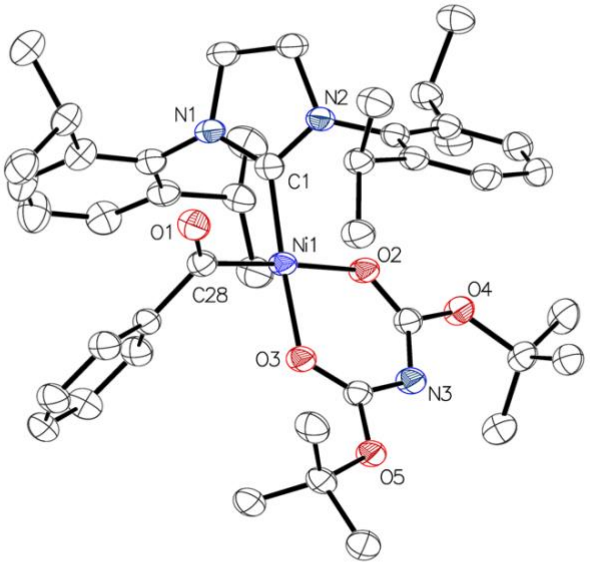

Figure S30. Solid-state structure of **8d** determined by SC-XRD. Anisotropic displacement ellipsoids drawn at the 50 % probability level. H atoms omitted for clarity. Figure generated with OLEX2.[18]

Table S10. Crystal data and structure refinement for **8d**.

Empirical formula	C44 H61 N3 Ni O5	
Formula weight	770.66	
Temperature	100.00(10) K	
Wavelength	1.54184 Å	
Crystal system	monoclinic	
Space group	*C*2/*c*	
Unit cell dimensions	*a* = 28.0052(3) Å	a = 90°
	b = 12.37220(10) Å	b = 117.1390(10)°
	c = 29.6294(3) Å	g = 90°
Volume	9135.89(17) Å^3	
Z	8	
Density (calculated)	1.121 Mg/m^3	
Absorption coefficient	0.945 mm^{-1}	
F(000)	3312	

Crystal color, morphology	yellow, block
Crystal size	0.151 x 0.124 x 0.099 mm^3
Theta range for data collection	3.352 to 80.276°
Index ranges	-35 < *h* < 34, -15 < *k* < 15, -34 < *l* < 37
Reflections collected	80239
Independent reflections	9848 [*R*(int) = 0.0412]
Observed reflections	8835
Completeness to theta = 74.504°	99.9%
Absorption correction	Multi-scan
Max. and min. transmission	1.00000 and 0.89808
Refinement method	Full-matrix least-squares on F^2
Data / restraints / parameters	9848 / 0 / 492
Goodness-of-fit on F^2	1.047
Final *R* indices [*I*>2sigma(*I*)]	*R*1 = 0.0390, *wR*2 = 0.0959
R indices (all data)	*R*1 = 0.0430, *wR*2 = 0.0980
Largest diff. peak and hole	0.291 and -0.459 e.$Å^{-3}$

Table S11. Atomic coordinates (x 10^4) and equivalent isotropic displacement parameters ($Å^2$x 10^3) for **8d**. U_{eq} is defined as one third of the trace of the orthogonalized U_{ij} tensor.

Atom	x	y	z	U_{eq}
Ni1	2941(1)	5674(1)	3939(1)	26(1)
O1	2516(1)	7053(1)	3115(1)	35(1)
O2	3334(1)	4514(1)	4394(1)	30(1)
O3	3406(1)	6697(1)	4426(1)	31(1)
O4	3937(1)	3674(1)	5037(1)	32(1)
O5	4182(1)	7270(1)	5058(1)	32(1)
N1	2008(1)	4653(1)	3124(1)	27(1)
N2	2774(1)	4125(1)	3183(1)	28(1)
N3	4035(1)	5514(1)	5021(1)	30(1)
C1	2543(1)	4755(1)	3396(1)	27(1)
C2	1861(1)	3809(1)	2731(1)	33(1)
C3	2381(1)	3665(1)	2695(1)	33(1)
C4	1624(1)	4981(1)	3291(1)	29(1)
C5	1217(1)	5700(1)	2985(1)	35(1)
C6	826(1)	5960(2)	3134(1)	47(1)
C7	839(1)	5530(2)	3568(1)	50(1)
C8	1241(1)	4818(1)	3863(1)	40(1)
C9	1642(1)	4520(1)	3733(1)	31(1)
C10	1189(1)	6180(1)	2502(1)	37(1)
C11	1152(1)	7413(2)	2496(1)	43(1)
C12	709(1)	5714(2)	2033(1)	46(1)
C13	2060(1)	3692(1)	4059(1)	33(1)
C14	2313(1)	3971(1)	4622(1)	44(1)
C15	1819(1)	2555(1)	3969(1)	48(1)
C16	3339(1)	3889(1)	3399(1)	28(1)
C17	3686(1)	4622(1)	3342(1)	30(1)
C18	4232(1)	4384(1)	3582(1)	33(1)

C19	4424(1)	3446(1)	3863(1)	36(1)
C20	4068(1)	2711(1)	3894(1)	35(1)
C21	3518(1)	2907(1)	3662(1)	31(1)
C22	3482(1)	5638(1)	3018(1)	30(1)
C23	3770(1)	6652(1)	3311(1)	37(1)
C24	3541(1)	5520(1)	2532(1)	42(1)
C25	3133(1)	2066(1)	3685(1)	34(1)
C26	3293(1)	1672(1)	4224(1)	40(1)
C27	3085(1)	1103(1)	3337(1)	41(1)
C28	2561(1)	6842(1)	3535(1)	28(1)
C29	2343(1)	7636(1)	3785(1)	29(1)
C30	2274(1)	8709(1)	3627(1)	35(1)
C31	2071(1)	9460(1)	3841(1)	41(1)
C32	1933(1)	9146(1)	4214(1)	40(1)
C33	2002(1)	8079(1)	4376(1)	39(1)
C34	2208(1)	7328(1)	4162(1)	34(1)
C35	3755(1)	4628(1)	4801(1)	27(1)
C36	3841(1)	6473(1)	4805(1)	28(1)
C37	4455(1)	3525(1)	5489(1)	31(1)
C38	4468(1)	2300(1)	5557(1)	40(1)
C39	4913(1)	3879(1)	5384(1)	34(1)
C40	4454(1)	4090(1)	5943(1)	36(1)
C41	4070(1)	8415(1)	4903(1)	33(1)
C42	3997(1)	8586(1)	4367(1)	39(1)
C43	3593(1)	8812(1)	4968(1)	37(1)
C44	4587(1)	8953(1)	5284(1)	40(1)

8.5 Solid-State Structure of 8i•C_6H_{14} *(CCDC 2285470)*

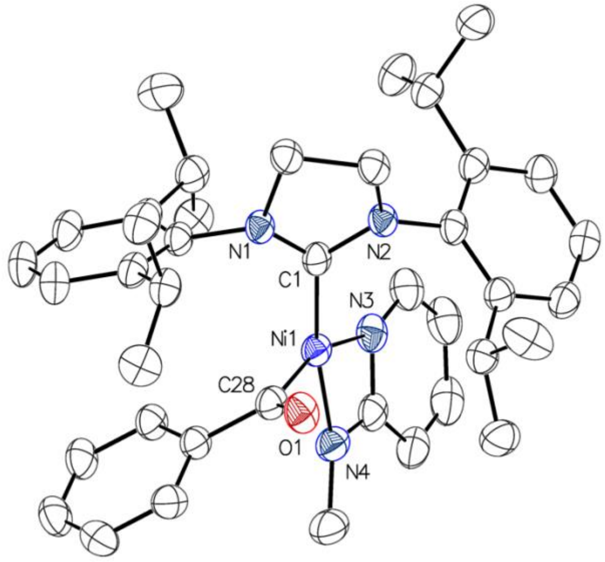

Figure S31. Solid-state structure of **8i** determined by SC-XRD. Anisotropic displacement ellipsoids drawn at the 50 % probability level. H-atoms and solvent molecules omitted for clarity. Figure generated with OLEX2.[18]

Table S12. Crystal data and structure refinement for **8i**•C_6H_{14}.

Empirical formula	C43 H57 N4 Ni O	
Formula weight	704.63	
Temperature	100.00(10) K	
Wavelength	1.54184 Å	
Crystal system	monoclinic	
Space group	*C*2/*c*	
Unit cell dimensions	*a* = 29.9671(3) Å	a = 90°
	b = 11.91190(10) Å	b = 119.846(2)°
	c = 26.8468(3) Å	g = 90°
Volume	8312.3(2) Å3	
Z	8	
Density (calculated)	1.126 Mg/m3	
Absorption coefficient	0.930 mm-1	
F(000)	3032	
Crystal color, morphology	orange-yellow, plate	

Crystal size	0.268 x 0.21 x 0.022 mm3
Theta range for data collection	3.401 to 80.640°
Index ranges	-38 < *h* < 38, -11 < *k* < 14, -34 < *l* < 34
Reflections collected	53382
Independent reflections	8928 [*R*(int) = 0.0433]
Observed reflections	7550
Completeness to theta = 74.504°	99.8%
Absorption correction	Multi-scan
Max. and min. transmission	1.00000 and 0.46248
Refinement method	Full-matrix least-squares on F^2
Data / restraints / parameters	8928 / 30 / 481
Goodness-of-fit on F^2	1.078
Final *R* indices [*I*>2sigma(*I*)]	*R*1 = 0.0432, *wR*2 = 0.1163
R indices (all data)	*R*1 = 0.0511, *wR*2 = 0.1216
Largest diff. peak and hole	0.368 and -0.511 e.Å^{-3}

Table S13. Atomic coordinates (x 10^4) and equivalent isotropic displacement parameters ($Å^2$x 10^3) for **8i•**C_6H_{14}. U_{eq} is defined as one third of the trace of the orthogonalized U_{ij} tensor.

Atom	x	y	z	U_{eq}
Ni1	6350(1)	6390(1)	5922(1)	30(1)
O1	6855(1)	5002(1)	6857(1)	43(1)
N1	6391(1)	7542(1)	6914(1)	31(1)
N2	6947(1)	8143(1)	6680(1)	31(1)
N3	6233(1)	7158(1)	5170(1)	36(1)
N4	6257(1)	5348(1)	5335(1)	38(1)
C1	6543(1)	7444(1)	6518(1)	30(1)
C2	6662(1)	8458(2)	7324(1)	36(1)
C3	7133(1)	8615(2)	7258(1)	35(1)
C4	5904(1)	7144(1)	6829(1)	32(1)
C5	5900(1)	6364(2)	7218(1)	35(1)
C6	5425(1)	5972(2)	7119(1)	42(1)
C7	4972(1)	6354(2)	6656(1)	46(1)
C8	4983(1)	7157(2)	6293(1)	41(1)
C9	5449(1)	7585(2)	6373(1)	34(1)
C10	6390(1)	5973(2)	7746(1)	37(1)
C11	6413(1)	4712(2)	7848(1)	49(1)
C12	6454(1)	6582(2)	8281(1)	46(1)
C13	5437(1)	8521(2)	5983(1)	38(1)

C14	5170(1)	8165(2)	5353(1)	45(1)
C15	5185(1)	9579(2)	6058(1)	55(1)
C16	7166(1)	8427(1)	6326(1)	32(1)
C17	7465(1)	7646(1)	6230(1)	34(1)
C18	7633(1)	7944(2)	5848(1)	39(1)
C19	7528(1)	8991(2)	5593(1)	40(1)
C20	7254(1)	9766(2)	5716(1)	38(1)
C21	7062(1)	9501(2)	6081(1)	34(1)
C22	7630(1)	6538(2)	6554(1)	39(1)
C23	7650(1)	5573(2)	6196(1)	52(1)
C24	8152(1)	6695(2)	7096(1)	60(1)
C25	6757(1)	10386(2)	6197(1)	37(1)
C26	6272(1)	10775(2)	5653(1)	47(1)
C27	7101(1)	11389(2)	6515(1)	52(1)
C28	6444(1)	5243(1)	6434(1)	34(1)
C29	5985(1)	4496(1)	6267(1)	33(1)
C30	6052(1)	3441(2)	6515(1)	38(1)
C31	5634(1)	2750(2)	6373(1)	44(1)
C32	5143(1)	3111(2)	5976(1)	46(1)
C33	5072(1)	4150(2)	5719(1)	42(1)
C34	5490(1)	4841(2)	5860(1)	36(1)
C35	6242(1)	6109(2)	4967(1)	38(1)
C36	6232(1)	5966(2)	4435(1)	47(1)

C37	6189(1)	6898(2)	4120(1)	54(1)
C38	6169(1)	7964(2)	4322(1)	50(1)
C39	6199(1)	8052(2)	4854(1)	43(1)
C40	6305(1)	4175(2)	5245(1)	50(1)
C41	6474(2)	1079(4)	4246(2)	51(1)
C42	6778(1)	2073(2)	4618(1)	47(1)
C43	7343(1)	2043(2)	4790(1)	46(1)
C41'	6383(6)	1360(19)	4183(14)	78(5)
C42'	6968(5)	1484(8)	4426(4)	60(2)
C43'	7204(2)	2460(9)	4860(4)	49(2)

9. Catalytic Cycle

A catalytic cycle summarizing the key observations and conclusions is depicted in Figure S32.

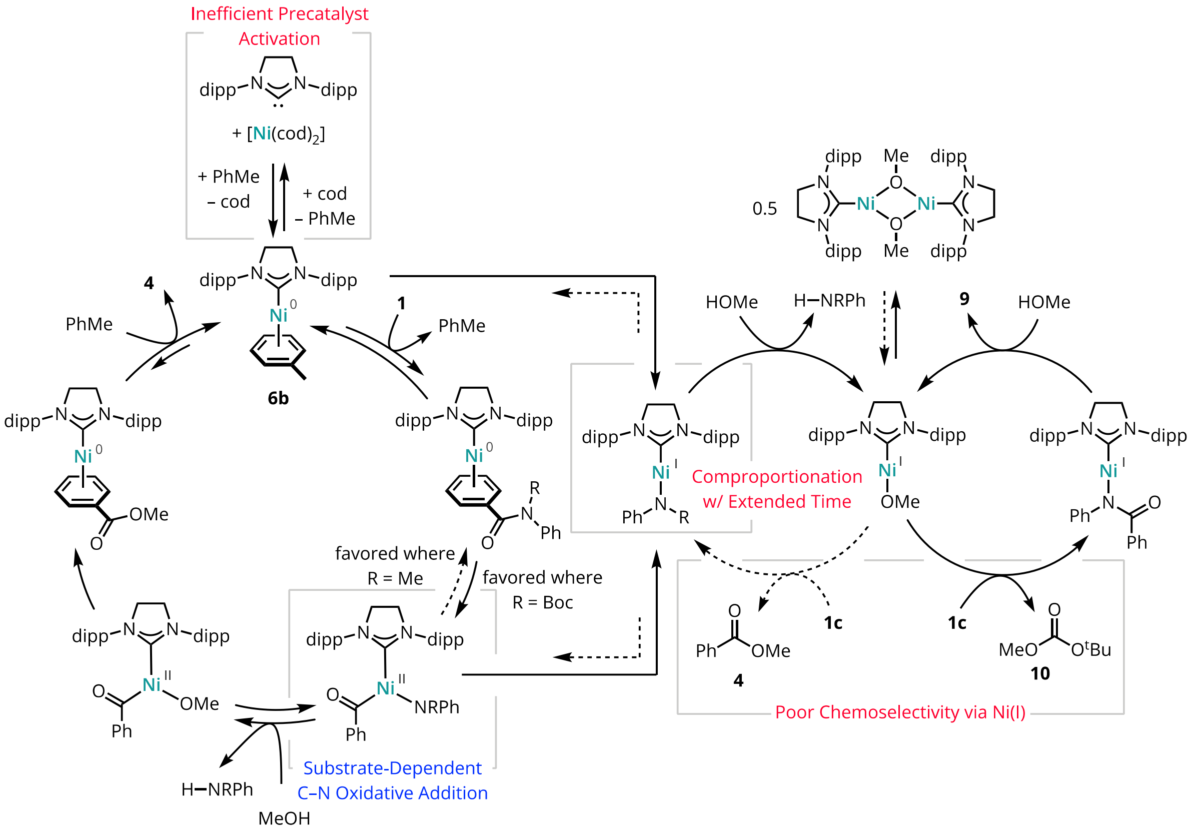

Figure S32. Catalytic cycle summarizing key observations and conclusions.

10. References

1. Borys, A. M. An Illustrated Guide to Schlenk Line Techniques. *Organometallics* **2023**, *42*, 182-196. DOI: 10.1021/acs.organomet.2c00535.
2. *Complete List of Prismacolor Colored Pencils.* https://www.jennyscrayoncollection.com/2020/04/complete-list-of-prismacolor- premier.html (accessed 2023-03-06).
3. Thomson, J. E.; Campbell, C. D.; Concellon, C.; Duguet, N.; Rix, K.; Slawin, A. M.; Smith, A. D. Probing the efficiency of N-heterocyclic carbene promoted O- to C-carboxyl transfer of oxazolyl carbonates. *J. Org. Chem.* **2008**, *73* (7), 2784-2791. DOI: 10.1021/jo702720a.
4. Hoshimoto, Y.; Hayashi, Y.; Suzuki, H.; Ohashi, M.; Ogoshi, S. One-Pot, Single-Step, and Gram-Scale Synthesis of Mononuclear [(η6-arene)Ni(N-heterocyclic carbene)] Complexes: Useful Precursors of the Ni0–NHC Unit. *Organometallics* **2014**, *33* (5), 1276-1282. DOI: 10.1021/om500088p.
5. Saper, N. I.; Hartwig, J. F. Mechanistic Investigations of the Hydrogenolysis of Diaryl Ethers Catalyzed by Nickel Complexes of N-Heterocyclic Carbene Ligands. *J. Am. Chem. Soc.* **2017**, *139* (48), 17667-17676. DOI: 10.1021/jacs.7b10537.
6. Pangborn, A. B.; Giardello, M. A.; Grubbs, R. H.; Rosen, R. K.; Timmers, F. J. Safe and Convenient Procedure for Solvent Purification. *Organometallics* **1996**, *15* (5), 1518–1520. DOI: 10.1021/om9503712.
7. Williams, D. B. G.; Lawton, M. Drying of Organic Solvents: Quantitative Evaluation of the Efficiency of Several Desiccants. *J. Org. Chem.* **2010**, *75* (24), 8351-8354. DOI: 10.1021/jo101589h.
8. Stoll, S.; Schweiger, A. EasySpin, a comprehensive software package for spectral simulation and analysis in EPR. *J. Magn. Reson.* **2006**, *178* (1), 42–55. DOI: 10.1016/j.jmr.2005.08.013.
9. Zhou, S.; Junge, K.; Addis, D.; Das, S.; Beller, M. A convenient and general iron-catalyzed reduction of amides to amines. *Angew. Chem. Int. Ed.* **2009**, *48* (50), 9507-9510. DOI: 10.1002/anie.200904677.
10. Meng, G.; Lei, P.; Szostak, M. A General Method for Two-Step Transamidation of Secondary Amides Using Commercially Available, Air- and Moisture-Stable Palladium/NHC (N-Heterocyclic Carbene) Complexes. *Org. Lett.* **2017**, *19* (8), 2158-2161. DOI: 10.1021/acs.orglett.7b00796.
11. Liu, Y.; Shi, S.; Achtenhagen, M.; Liu, R.; Szostak, M. Metal-Free Transamidation of Secondary Amides via Selective N-C Cleavage under Mild Conditions. *Org. Lett.* **2017**, *19* (7), 1614-1617. DOI: 10.1021/acs.orglett.7b00429.
12. Luo, Z.; Xiong, L.; Liu, T.; Zhang, Y.; Lu, S.; Chen, Y.; Guo, W.; Zhu, Y.; Zeng, Z. Palladium-Catalyzed Decarbonylative Suzuki-Miyaura Coupling of Amides To Achieve Biaryls via C-N Bond Cleavage. *J. Org. Chem.* **2019**, *84* (17), 10559–10568. DOI: 10.1021/acs.joc.9b01103.
13. Wu, H.; Guo, W.; Daniel, S.; Li, Y.; Liu, C. Fluoride-Catalyzed Esterification of Amides. *Chem. Eur. J.* **2018**, *24* (14), 3444–3447. DOI: 10.1002/chem.201800336
14. Hie, L.; Fine Nathel, N. F.; Shah, T. K.; Baker, E. L.; Hong, X.; Yang, Y. F.; Liu, P.; Houk, K. N.; Garg, N. K. Conversion of amides to esters by the nickel-catalysed activation of amide C-N bonds. *Nature* **2015**, *524* (7563), 79-83. DOI: 10.1038/nature14615
15. *CrysAlisPro, version 171.42.80a*; Rigaku Corporation: Oxford, UK, 2023. (accessed.
16. Sheldrick, G. M. SHELXT, version 2018/2. *Acta. Crystallogr.* **2015**, *A71*, 3–8.
17. Sheldrick, G. M. SHELXL, version 2019/2. *Acta. Crystallogr.* **2015**, *C73*, 3–8.
18. Dolomanov, O. V.; Bourhis, L. J.; Gildea, R. J.; Howard, J. A. K.; Puschmann, H. Olex2, version 1.5. *J. Appl. Cryst.* **2009**, *42*, 339-341.
